# Capture and return of sexual genomes by hybridogenetic frogs provide clonal genome enrichment in a sexual species

**DOI:** 10.1038/s41598-021-81240-5

**Published:** 2021-01-15

**Authors:** Marie Doležálková-Kaštánková, Glib Mazepa, Daniel L. Jeffries, Nicolas Perrin, Marcela Plötner, Jörg Plötner, Gaston-Denis Guex, Peter Mikulíček, Albert J. Poustka, Jose Grau, Lukáš Choleva

**Affiliations:** 1 Laboratory of Fish Genetics, Institute of Animal Physiology and Genetics CAS, v. v. i., 277 21 Libechov, Czech Republic; 2 Department of Ecology and Evolution, University of Lausanne, Biophore, 1015 Lausanne, Switzerland; 3 Department of Ecology and Genetics, Evolutionary Biology, Norbyvägen 18D, , 75236 Uppsala, Sweden; 4 Museum Für Naturkunde, Leibniz-Institut Für Evolutions- Und Biodiversitätsforschung, Invalidenstraße 43, 10115 Berlin, Germany; 5 Private Laboratory, Dätwil, Hauptstrasse 2, 8452 Adlikon Zürich, Switzerland; 6 Department of Zoology, Faculty of Natural Sciences, Comenius University in Bratislava, Ilkovicova 6, 84215 Bratislava, Slovak Republic; 7 Max Planck Institute for Molecular Genetics, Evolution and Development Group, Ihnestrasse 73, 14195 Berlin, Germany; 8 Dahlem Centre for Genome Research and Medical Systems Biology, Max-Planck-Straße 3, 12489 Berlin, Germany; 9 Services in Molecular Biology GmbH, Rudolf-Breitscheid-Str. 70, 15562 Rüdersdorf, Germany; 10 Department of Biology and Ecology, Faculty of Science, University of Ostrava, 701 03 Ostrava, Czech Republic

**Keywords:** Genetics, Molecular biology, Zoology

## Abstract

Hybridogenesis is a reproductive tool for sexual parasitism. Hybridogenetic hybrids use gametes from their sexual host for their own reproduction, but sexual species gain no benefit from such matings as their genome is later eliminated. Here, we examine the presence of sexual parasitism in water frogs through crossing experiments and genome-wide data. We specifically focus on the famous Central-European populations where *Pelophylax esculentus* males (hybrids of *P. ridibundus* and *P. lessonae*) live with *P. ridibundus.* We identified a system where the hybrids commonly produce two types of clonal gametes (hybrid amphispermy). The haploid *lessonae* genome is clonally inherited from generation to generation and assures the maintenance of hybrids through a process, in which *lessonae* sperm fertilize *P. ridibundus* eggs. The haploid *ridibundus* genome in hybrids received from *P. ridibundus* a generation ago, is perpetuated as clonal *ridibundus* sperm and used to fertilize *P. ridibundus* eggs, yielding female *P. ridibundus* progeny. These results imply animal reproduction in which hybridogenetic taxa are not only sexual parasites, but also participate in the formation of a sexual taxon in a remarkable way. This occurs through a process by which sexual gametes are being captured, converted to clones, and returned to sexual populations in one generation.

## Introduction

Hybridogenesis is an extraordinary mode of reproduction, comprising components from both asexual and sexual reproduction^1^. This mode of reproduction is usually found in hybrids arising from historical or ongoing hybridization events. Together with two other modes of unusual reproduction, parthenogenesis and gynogenesis^2–5^, it has the potential to clonally perpetuate the parental genomes^6^, and maintain an F_1_ hybrid genotype. While parthenogenesis and gynogenesis are fully clonal modes of reproduction, hybridogenesis is referred to as hemiclonal reproduction. It involves the selective transmission of one of the parental genomes, while the other is excluded from the germline and renewed by mating with the sexual host species^1,7^. As a consequence, hybridogenetic organisms are often considered sexual parasites. They use gametes from their sexual hosts for their own reproduction, while their sexual hosts gain no benefit from matings with hybridogenetic individuals, as their genome will be eliminated during gametogenesis and, therefore, will not contribute to future generations^7^.

The eliminated genome is usually received as a recombinant gamete from the previous generation of the sexual host^8,9^ while the transmitted genome is clonal; it fuses with the recombinant gamete resulting in the generation of a new hybrid^10,11^. The elimination process is assumed to maintain the integrity of parental genomes, which is the main requirement for hemiclonal inheritance^12^.

While clonally reproducing females use several reproductive strategies to form new single-sex generations of female progeny^1,3,13,14^, cases of clonal reproduction resulting only in hybrid males are rare^10^. Assumed reasons for their rarity are lower viability and a high level of sterility^15,16^. Existing evolutionary models predict that such systems would be unstable and go extinct^17–19^. However, some examples of stable all-male hybrid systems have been reported^6,11,20–22^, which raises the question of how these systems remain evolutionarily stable.

The European *Pelophylax esculentus* complex is the best-studied model of hybridogenesis. It involves two parental species, the pool frog *P. lessonae* (LL) and the marsh frog *P. ridibundus* (RR), and their hybrid the edible frog *P. esculentus* (RL)*. Pelophylax esculentus* can be produced as diploid or triploid individuals of both sexes, they can also vary in their patterns of gamete production and the population assemblage. In the so-called L-E system, *P. esculentus* is usually represented by both sexes, clonally transmitting the *ridibundus* genome to gametes, and re-incorporating the *P. lessonae* genome via mating with sympatric *P. lessonae* individuals^23^. In some cases, matings between two hybrids resulting in *P. ridibundus* progeny also occur^24–26^, however, mostly inviable or infertile, presumably due to the accumulation of deleterious mutations in clonal genomes^27–31^. For the functional survival of the so-called R-E system, hybrids pass on the *lessonae* genome and parasitize sympatric *P. ridibundus* individuals to re-incorporate the *ridibundus* genome each generation. Interestingly, typical diploid R-E populations contain only male *P. esculentus* transmitting mostly the *lessonae* genome with cases of the *ridibundus* genome as well^22,32–35^. The inheritance pattern in water frogs is consistent with an XX-XY sex determination system, and males are heterogametic^22,23^.

We report a mechanism of animal reproduction in general, and hybridogenesis in particular, with significant consequences to the reproduction of a host sexual species. In this system, hybrid males sexually parasitize a sexual host and capture the maternal genome for one generation. The captured genomes are then returned in a clonal form back to the population of the sexual host via the production of sexual daughters. Our study system includes hybridogenetic *P. esculentus* hybrids, which are exclusively males competing with sexual *P. ridibundus* males for matings with *P. ridibundus* females. Combining laboratory crossing experiments and genetic analysis of produced gametes and resulting offspring, we show that the maternal genomes are not systematically excluded from the germline. Instead, both maternal and paternal genomes are transmitted from one generation to another, maternal into sexual daughters and paternal into hybrid sons. These results indicate that such an unorthodox reproductive system is more common in water frogs than previously thought. We discuss the putative impact of amphispermic hybridogenesis on population dynamics, as well as the molecular mechanisms of this reproductive strategy of sexual parasites.

## Results

### Microsatellite taxon assignments

**Figure 1:**
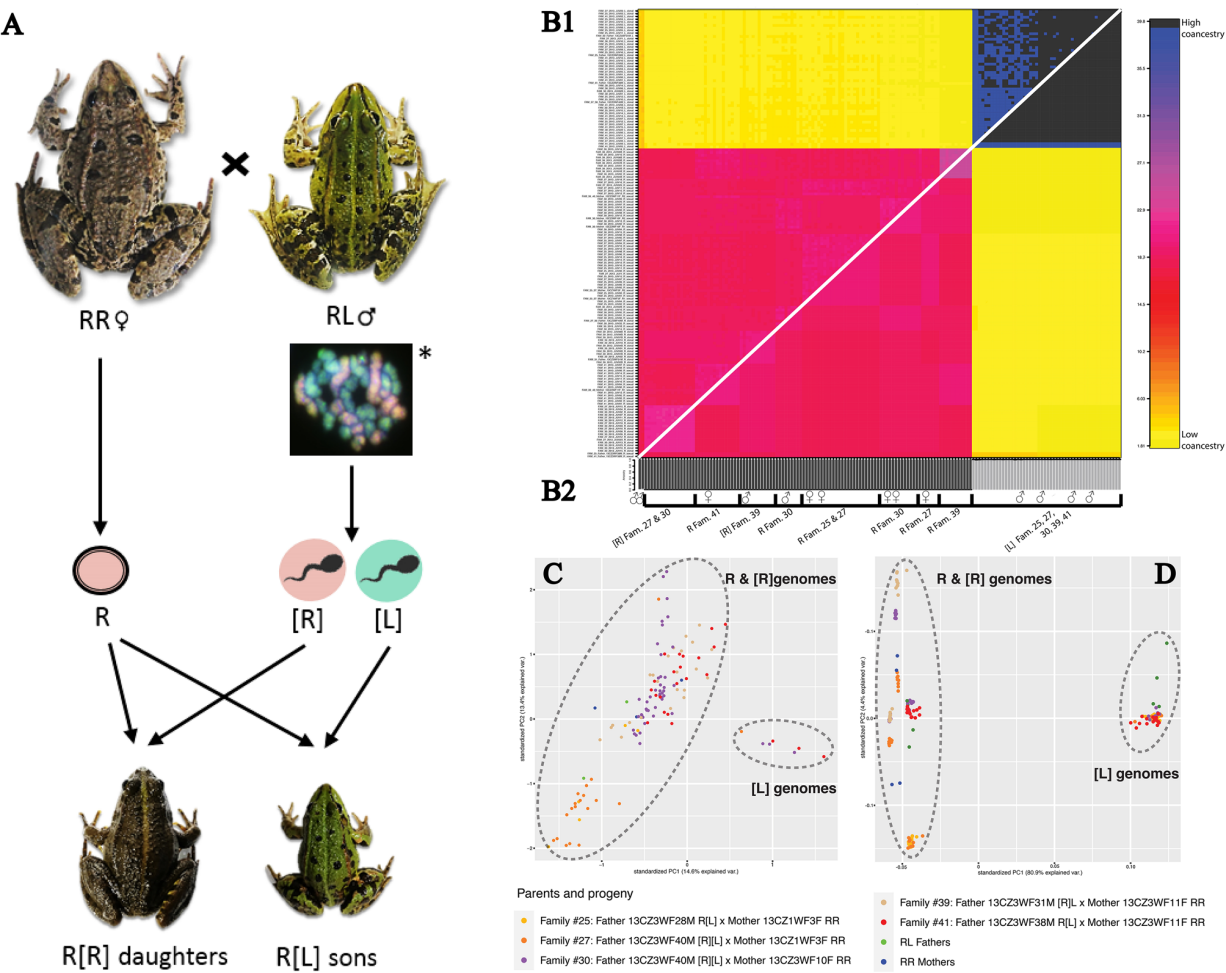
Genetic identity of the phased *Pelophylax* genomes based on 81 individuals and five families. RR denotes *P. ridibundus* mothers and daughters, RL denotes *P. esculentus* fathers and sons. R denotes sexual genomes, [R] and [L] denote clonal genomes. (**A**) An illustration of the laboratory crossing experiments that resulted in R[R] daughters and R[L] sons; photos show real individuals participating in the experiment; * late prophase I of the *P. esculentus* male´s first meiotic division using comparative genomic *in-situ* hybridization with *lessonae* specific probes (red color) and *ridibundus* specific probes (green color); see Doležálková et al.^34^. (**B1**) fineRADstucture heatmap of haplotypes similarity (2219 SNPs): co-ancestry matrix based on a RADseq dataset, above the diagonal are the individual scores, below are the population averages. The color scale legend on the right indicates the relatedness between haploid genomes. A cluster of clonal [L] genomes is apparent in the upper right of the graph (black/dark blue color), while sexual R and clonal [R] on the down left (rose/red color). (**B2**) STRUCTURE ancestry bar plots of the respective individuals, Q-value scale indicated on the left. Phased haploid genomes of mothers are indicated with ♀ and fathers with ♂, other genomes representing progeny with families´ membership indicated with frames. (**C**) Principal component analysis (PCA) of phased microsatellite data based on 15 loci. (**D**) Results of PCA based on phased genotypes of ddRADseq data (2219 SNPs). The clusters determine sexual R/clonal [R] genomes and clonal [L] genomes. Each point represents an individual haploid multilocus genotype; one color and symbol denote one family.

Experimental crosses between *P. esculentus* males and *P. ridibundus* females (Fig. 1A) resulted in 34 families and 523 juveniles that reached metamorphosis (Supplementary Table S1). Microsatellite analyses performed on 18 parental individuals and 220 progeny across 17 families with metamorphosed juveniles detected a total of 71 alleles, from which eight were known as *lessonae*-specific and 63 alleles as *ridibundus*-specific (Table 1). Microsatellites supported the taxon assignments of adults based on morphology and further assigned RR/RL genotypes to 60/160 juveniles, respectively (Table 1; Supplementary Tables S2 and S3). The presence of one or two alleles per locus together with allele peak sizes (dosage effect) suggested that all individuals were diploid. Allele-dosage effects as indications of polyploidy were not observed. Nine out of 12 hybrid males had only sons (always of RL genotypes). Two males (M4, M11) had mixed progeny of daughters (always of RR genotypes) and sons (always of RL genotypes) with sex ratios of approximately 1:1. Hybrid male M12 had only three RR daughters (Table 2). Four juveniles had no fully developed gonads making it impossible to determine their sex.

**Table 1:** Microsatellite loci used in this study.

Locus	*lessonae*-specific alleles	*ridibundus*-specific alleles
RICA2a34^1^	144	–
RICA5^2^	262	–
Rrid013A^2,3^	301	287/293
Ga1a19^4^	197	203/207/211/224/245/249/251
RICA18^2^	188	–
RICA1b5^2^	120	134/136/140
Res14^5^	140	146/150
Res20^5^	124	–
Re1Caga10^4^	–	106/108/110/113/115/117/119/122/127/133/137
Re2Caga3^4^	–	173/204/212/223/232/234/236/239
RICA1b6^4^	–	77/80/83/88/90/96/98/103
Res22^5^	–	85/106/108/113/130
Rrid059A^1^	–	129/131/135/137/142
Rrid082A^1^	–	163/169/178/182/184
Rrid169A^1^	–	187/189/192/195/197/204/208

The allele sizes are given in base pairs.

^1^Christiansen and Reyer^48^; ^2^Garner et al.^64^; ^3^Hotz et al.^65^; ^4^Arioli et. al^66^; ^5^Zeisset et al.^67^.

**Table 2:** Inheritance of the parental genome(s) by *P. esculentus* males and sex in B1 progeny.

Cross ID	Male	Gamete	RR progeny			RL progeny		
			**♂**	**♀**	Undev	**♂**	**♀**	Undev
25-2013	M1	[L]	–	–	–	20	–	–
32-2013	M1	[L]	–	–	–	3	–	–
65-2013	M3	[L]	–	–	–	1	–	–
49-2013	M4	[L], [R]	–	1	–	1	–	–
39-2013	M4	[R]	–	22	–	–	–	–
42-2013	M5	[L]	–	–	–	64	–	–
53-2013	M8	[L]	–	–	–	2	–	–
41-2013	M9	[L]	–	–	–	20	–	–
57-2013	M10	[L]	–	–	–	3	–	–
66-2013	M10	[L]	–	–	–	20	–	2
27-2013	M11	[L], [R]	–	14	1	11	–	–
30-2013	M11	[L], [R]	–	28	–	21	–	–
59-2013	M12	[R]	–	2	1	–	–	–
60-2013	M13	[L]	–	–	–	19	–	–
54-2013	M14	[L]	–	–	–	2	–	–
63-2013	M14	[L]	–	–	–	3	–	–
26-2013	M16	[L]	–	–	–	17	–	–

Each *P. esculentus* male (M1–M16) was crossed with two *P. ridibundus* females, resulting in either *P. ridibundus* (RR) or *P. esculentus* (RL) progeny. Progeny was determined with microsatellites. Undev = juveniles with undeveloped gonads and unknown sex; [R] = clonal *ridibundus* genome; [L] = clonal *lessonae* genome.

### Inheritance and relatedness of parental genomes

Here, we examined the heredity modes of RL hybrid males concerning the fate of their L and R genomes. We assumed that RL males are fixed F1´s and, from a definition of hybridogenesis, eliminate one parental genome from their germline and transfer only the second parental genome to gametes. To do so, we designed methodological approaches allowing us to trace DNA variants of progeny backward to their parentals and separated a genome of the father from that of the mother. Phased haploid genomes were then analyzed for their relationships within families, taxon assignments, and levels of recombination using Principal component analysis (PCA), Principal coordinate analysis (PCoA), fineRADstucture heatmap, and STRUCTURE ancestry bar plots. We denoted sexual genomes of *P. ridibundus* as R and of *P. lessonae* as L, the hybridogenetic hybrid *P. esculentus* as RL‚ and the clonally inherited genomes as [R] and [L].

GenAlEx estimated a total of 204 multilocus genotypes (MLGs) based on microsatellite profiles of 220 phased offspring. A total of 11 MLGs were found within *lessonae* genomes of 160 RL sons. A total of 193 MLGs were found within *ridibundus* genomes of 160 RL sons and 60 RR daughters. Inspecting the allelic variability by filtering out variation caused by missing data showed RL sons shared the same MLG. This indicates the clonal inheritance of the L genome (further [L]) in all but one RL son (60-2013 JUV19), which inherited the R-specific allele at locus Re1Caga10 from the father (Supplementary Figures S1 and S2). In contrast to hybrid sons, the paternal *ridibundus* genomes in RR daughters exhibited three MLGs, corresponding to the allelic profiles of three *P. esculentus* fathers. Daughters and fathers shared the same MLG within each family, indicating that R genomes (further [R]) were inherited clonally as well (Supplementary Figure S1). PCoA run in GenAlEx and PCA performed with R package *radiator* supported two groups of microsatellite MLGs; the two principle coordinates accounted for 65.16%/14.60% (GenAlEx/*radiator*) for axis 1 and 10.26%/13.40% (GenAlEx/*radiator*) for axis 2 of genetic variation (Fig. 1C; Supplementary Figure S3). One cluster grouped *ridibundus*-specific MLGs of adult and juvenile *P. esculentus* and *P. ridibundus*, with *lessonae-*specific MLG of adult and juvenile *P. esculentus*.

ddRADseq analysis was run on 81 individuals from five families and 2,265 informative single nucleotide polymorphisms (SNPs) (Fig. 1B1,B2,D). PCA was first performed on unphased diploid genotypes and distinguished RR from RL individuals (not shown). The shared father of the families 27- and 30- simultaneously passed [R] and [L] genomes, fathers of the families 25- and 41-2013 passed only the [L] genome‚ and the father of the family 39-2013 passed only the [R] genome (Fig. 1D). Of the 15,676 SNPs called from the RADseq data, 2219 could be phased and were present in at least both parents and 75% of offspring in one or more families. The dendrogram of phased haplotypes showed more variability in maternal recombined genomes than in [R] and [L] genomes inherited from *P. esculentus* fathers (Supplementary Figure S4). PCA was used again to summarise this phased dataset and discriminated between the L and R genomes. PC 1 (78,60% in *radiator*), PC 2 (3,10%) and PC 3 captured the variation among the sexual R and clonal [R]/[L] genomes and were optimal for differentiating family-wise R-sexual and [R]-clonal genomes. We were able to calculate linkage maps using conventional software for males and females (Supplementary Figure S2), even though algorithms of both Lep-Map3 and MSTmap are not designed for data with non-Mendelian inheritance patterns. In general, we revealed a complete linkage of loci within the germline of RL males. The linkage maps of RR females consisted of several linkage groups of different lengths, indicating functional crossing-over and recombination (Supplementary Figure S2).

## Discussion

Our study shows that asexual organisms possess much higher reproductive variation than previously thought. The results of genetic analyses carried out on the basis of SNP markers and microsatellites indicate the absence of recombination between the L and R genomes in *P. esculentus* males. We could not exclude, however, an unrecognized leakage of subgenomic DNA due to general marker limitations. Among other asexual taxa, hybridogenetic *P. esculentus* males are exceptional in their ability to generate individuals of their parental species (Fig. 1A) in relatively substantial numbers. More importantly, this reproductive strategy indicates mechanisms of non-Mendelian inheritance that may change our view of the role of asexuality in the dynamics of mixed population systems in which most asexuals exist.

### Clonal inheritance of both paternal genomes

Both the microsatellite and fineRADstructure analyses, and PCA of the phased genomes, revealed the variation of inheritance patterns in *P. esculentus*‚ allowing fathers to produce not only clonal [L] but also [R] gametes, or both (Fig. 1; Supplementary Figure S3). Allelic profiles compared between mother and offspring verified the Mendelian inheritance (i.e., with recombination) of R genomes coming from *P. ridibundus* females (Supplementary Figure S1). The comparison of male and offspring haplotypes, however, showed the complete physical linkage between markers in haploid [L] and [R] genomes from *P. esculentus* males, indicating their non-Mendelian inheritance from the father.

The level of relatedness between the clonal [L] genomes was similar among individuals and might represent a single hemiclonal lineage. Indeed, allelic variation compared with previously analyzed hybrid males^35^ supported the sharing of *lessonae*-specific alleles within the [L] genomes, containing a Y chromosome or at least a male sex-determining region. Clonal [R] gametes from fathers and sexual R gametes from the mothers were characterized by low divergence. Clustering of the phased genomes showed a pattern in which a terminal branch length varied between the sexual R genomes while clonal ones were characterized by a much shorter branch length (Supplementary Figure S4).

Our data supported the absence of recombination between R and L genomes except one *P. esculentus* offspring, which inherited a single R microsatellite allele instead of an expected [L] allele from its father. Traces of occasional introgression, called genome leakage, have already been identified in water frogs^22,35–37^. The recent example of introgression has been linked to unexpected paternal genomic elements in the celibate genome^38^. Whether the recombination of *P. esculentus* offspring was caused by disturbed segregation during meiosis, intrachromosomal recombination, or through paternal genomic elements remains unclear.

### Hybrid amphispermy as a reproductive strategy

The phenomenon of hybrid amphispermy, when even a single *P. esculentus* male can form sperm with clonal genomes of two different species, seems to be widespread^22,32,39–43^. A similar strategy is assumed in the Australian carp gudgeons, which also exhibit hybridogenetic heredity of both parental genomes, although the pattern has not been observed at the individual scale; a male-biased group clonally inherits the paternal genome, while a female-biased group, clonally inherits the maternal genome^11^.

In our study, at least one-quarter of water frog hybrids formed both types of progeny, *P. esculentus* sons, and *P. ridibundus* daughters. Besides a pattern confirming an XY sex determination system, the total number of *P. ridibundus* daughters produced by hybrid fathers was quite impressive: 67 out of 274 offspring. Previous experimental data support a broader scale of the phenomenon in which even one-third of progeny were *P. ridibundus* females^22^. In the case of vertebrate systems that mirror hybrid female asexuality, one would assume a selective evolutionary preference for males to produce 100% hybrid progeny, providing them a two-fold reproductive advantage compared with sexual propagation^44^.

Rather than a failure of a proper genome elimination of one parental genome, we propose that *P. esculentus´* male amphispermy is a result of an evolutionary strategy providing benefits to asexual males in coping with mating challenges. Sperm-dependent asexuals may face some degree of sperm-limitation^2^. In spite of this, asexual females sharing sexual males do not need to produce males on their own as sexual males are usually willing to spawn repeatedly during the whole reproductive season. The opposite situation can be expected in the R-E system in which *P. esculentus* males compete with *P. ridibundus* males for *P. ridibundus* females. The ovarian cycle of water frog females in the temperate zone of Europe provides a finite number of oocytes that will be ovulated in the next spawning season^45^. Sexual females are thus a much more limited source of mates than sexual males. Under such circumstances, the production of *P. ridibundus* daughters by *P. esculentus* males may increase the number of females in mixed populations and, in turn, the chance of hybrid males to successfully find a mate.

### Genomes jumping from sex to asex and back to sex

Our crossing experiments allowed us to investigate a remarkable mechanism of inheritance operating through the interaction between both non-sexual hybrids and sexual species. *Pelophylax esculentus* males themselves were originally formed through the fusion of ancestral R gametes provided by *P. ridibundus* and ancestral L gametes obtained from *P. lessonae*, both representing species with Mendelian inheritance. However, once trapped within a hybrid, the [R] and [L] genomes usually do not recombine during spermatogenesis. The recombinant R gamete from a sexual species is assumed to be removed during the DNA elimination process in the hybrid germline, and in this way to be permanently lost from the sexual population. According to current data, the recombinant R genome is only temporarily misplaced from the sexual population rather than eliminated. In fact, the R genome is essentially ´frozen´ in some *P. esculentus* males for a single generation before it is returned, through non-Mendelian inheritance, back to the sexual population in the form of *P. ridibundus* females. Presumably, as the non-Mendelian inheritance of these R genomes has only persisted for one generation, deleterious effects are negligible. Therefore, we might expect the fitness of these females to be comparable to those of homotypic crosses, although this fate remains unexplored.

### Concluding remarks and outlook

Many asexual hybrids are reproductively dependent on sexual partners and can, therefore, be viewed as sexual parasites^3^. Despite the hundreds of crossing experiments investigating the inheritance of hybrids conducted in the past, the reproductive strategies of hemiclonal hybrids are not fully understood. A characteristic feature of hybridogenetic taxa is that one parental genome is perpetuated by hybrids in a clonal form, while the other is recombined in each generation by the Mendelian parental species. Whether the ability of *P. esculentus* males to form new sexual females is beneficial or rather disadvantageous for the hybrids‚ as the females are produced at the expense of hybrid genotypes, remains unknown. We hypothesize, however, that this may represent a way for hybrid males to increase the number of potential female partners in the population, and thus, a so-far unexplored strategy of asexual vertebrates to maintain themselves. Our study adds another aspect to the phenomenon related to sexual populations. If standard, a mechanism affecting the R genome that is captured, made clonal, then returned to the sexual population, may theoretically slow down a recombination-driven evolution of parasitized *P. ridibundus* populations in comparison to pure sexual populations with fully Mendelian inheritance. This situation suggests sexual parasites have outstanding potential to change the evolutionary trajectory of their host species.

## Material and methods

### Sampling and crossing experiment

A total of 26 adult water frogs that originated from three mixed R-E and two pure *P. ridibundus* populations were included in this study (Table 3; Supplementary Table S2). Each of 16 *P. esculentus* males was crossed with two or three of nine *P. ridibundus* females to explore their gamete production (Supplementary Table S1). One *P. ridibundus* male, as a control, was crossed with all females. Crossing experiments followed Berger et al.^46^ with modifications following Pruvost et al.^47^. Fully metamorphosed froglets were anesthetized in a 2 mg/l MS-222 solution and dissected for a morphological sex determination.

**Table 3:** Summary of adult *Pelophylax* individuals used in this study.

Population	N	Sample site	Latitude, longitude	Sex	nuDNA
R-E	1	Albrechtičky	49.70805 N, 18.09888 E	M	RL
	1	Dolní Benešov	49.91222 N, 18.12000 E	F	RR
	4	Košatka	49.73611 N, 18.15416 E	F	RR
	1	Košatka	49.73611 N, 18.15416 E	M	RR
	15	Košatka	49.73611 N, 18.15416 E	M	RL
R	3	Cítov	50.36638 N, 14.44694 E	F	RR
	1	Liběchov	50.41012 N, 14.45194 E	F	RR

R-E, *P. ridibundus*-*P. esculentus* population; R, pure *P. ridibundus* population; RR, *P. ridibundus*; RL, *P. esculentus.*

### Molecular labwork

DNA was extracted using the NucleoSpin DNA extraction kit (Macherey-Nagel, Düren, Germany). A total of 246 individuals (26 adults, 220 juvenile frogs) were genotyped at 15 microsatellite loci after Christiansen and Reyer^48^ using two multiplex PCR sets. Multiplex 1 for loci: Res20, RlCA1b5, RlCA5, RICA18, Ga1a19, RICA2a34, Rrid013A and Res14. Multiplex 2: Res22, Rrid059A, Rrid169A, Re1Caga10, Re2Caga3, RlCA1b6 and Rrid082A (Supplementary Table S4). These loci have the power to distinguish taxa of the Central-European water frogs^47^. Fragment-length analyses were performed on an ABI 3730 Avant capillary sequencer (Applied Biosystems, Foster City, California, USA) with an internal size standard (GeneScan-500 LIZ, Thermo Fisher Scientific, Waltham, MA, USA).

The detection of SNPs was based on the double digest restriction-site associated DNA (ddRADseq) approach. For comparative analysis of high-density SNP data, we chose 81 individuals from five families (25-, 27-, 30-, 39-, and 41-2013), which differed in the genome clonally passed down by the parents. We scanned genomes for SNPs data of both parental genomes. ddRADseq libraries were prepared following the protocol of Peterson et al.^49^ with modifications by Brelsford et al.^50^. Briefly, template DNA for each sample at equimolar concentrations (approx. 15 ng/μl) was digested using a combination of *MspI* and *SbfI* restriction enzymes. P1 and P2 adapters containing barcodes were then ligated‚ and these ligated fragments were amplified in four PCR replicates of 20 cycles, lowering the risk of polymerase-induced errors. The products were then pooled, and fragments of 350–500 bp were selected from 2% agarose gels, of which 135–139 bp belonged to the pair of Illumina primers, adaptors, and barcodes. The pool of 96 individual libraries was sequenced with a single-end run of 100 cycles (for 150 bp) on a HiSeq 2500 Illumina instrument at the Genomic Technologies Facility at the University of Lausanne, Switzerland.

### Analyses of genome inheritance with phasing variants in hybrids

The microsatellite alleles were scored with GeneMapper v. 3. 7 (Applied Biosystems, Zug, Switzerland). Raw genotypes of sexual species were checked for genotyping errors and null alleles with Micro-Checker v. 2.2.3^51^. Hybrid genotypes were converted to separate *lessonae-* and *ridibundus*-specific alleles, following Doležálková-Kaštánková et al.^35^. To distinguish clonally inherited and recombined genomes in backcrossed B1 individuals, we ran the analysis to separate multilocus genotypes (MLGs) using GenAlEx v. 6. 41^52^. To reveal potential gene flow between L and R genomes, we analyzed allele frequencies, heterozygosity, and polymorphism using GenAlEx v. 6. 41^52^. We performed PCoA in GenAlEx v. 6. 41^52^ and, in parallel, PCA in *radiator* v 0.0.18 R package^53^ with data standardization, which was based on the total variation of microsatellite allele frequencies. MLGs with more than 65% of missing data were removed from the analysis.

ddRADseq reads were demultiplexed with the STACKS v 1.42 module^54^ process_radtags (–renz_1 sbfI –renz_2 mspI -E phred33 -r -c -q -i fastq –adapter_1 ACACTCTTTCCCTACACGACGCTCTTCCGATCT –adapter_2 GTGACTGGAGTTCAGACGTGTGCTCTTCCGATCT –adapter_mm 2). To clip the adaptor sequence fragments, if present, we applied a custom script (Supplementary File S1). We generated 105,780,761 single-end reads of average read quality > 35, and after applying processing with the Stacks pipeline, 15,676 SNPs remained. Of these, 2265 were informative and were thus retained for the subsequent analyses. The *Pelophylax lessonae* draft genome assembly (3,453,250 scaffolds) was prepared for mapping purposes with a custom awk script, connecting series of 450 sequential scaffolds (> 190 bp) into an artificial sequence spaced with the fragments of 300 N’s. Such an artificially stitched reference genome contained 4,605 scaffolds that were used for further mapping. The reads were mapped on a draft genome assembly of *P. lessonae* with *bowtie2* (–local –very-sensitive), the mapped data were filtered with samtools v 0.1.19 for the mapping quality > 20. Further, we constructed a common catalog in Stacks v 1.42 using the *ref_map.pl* pipeline. Genotypes were outputted with the Stacks module *populations* treating each family separately by specifying a family-specific “population map “ file and keeping parameters –p = 2 and –r = 0.95, having aimed to retain only the loci that were present in both parents and > 95% of the offspring in a given family. Each population catalog was whitelisted for the biallelic loci only. Ultimately, the five whitelists were merged into one (comprising 4,755 loci), and the *population's* module was rerun on the initial catalog for all five families together.

So far, known scripts phase parent-progeny duos and trios on data coming from model Mendelian species like humans and imply imputation, i.e., the probability of the mistake is calculated for a missing allele in a genotype, and, if it does not follow the Mendelian pattern, it is considered as an artifact of the data. We designed a script that separates non-Mendelian hybrid profiles to haploid ones according to species-specificity of alleles or SNP sites in parental genomes. Using the 15,676 SNP data set for five families, the R genomes were separated from L genomes using custom python scripts. Loci were phased based on their segregation patterns among mother, father, and offspring, separately for each family. For example, an R specific allele will be heterozygous in the father, homozygous in the mother, and heterozygous in all offspring of an R[L] x RR cross. The [L] allele, in this case, is the one which is present only in the father and the offspring, but not in the mother. Similar tests were performed and tailored specifically for all cross types. Such tests can be sensitive to data artifacts. For example, null alleles in one parent, so that the progeny would be heterozygous (null-allele/allele), while appearing as a homozygous. These artifacts were checked for in our scripts, and where appropriate, these loci were removed. For a detailed explanation of this phasing approach and all code used, see https://github.com/DanJeffries/Hybridogen_paper.

### Analyses of the structure and relatedness between clonal and sexual haploid genomes

STRUCTURE analysis (v 2.3.4)^55^ with K = 2 was made by running a chain of 30,000 with burn-in 10,000 under the admixture model with correlated allele frequencies of microsatellites. The alpha parameter was set to 0.5 following recommendations of Wang^56^, who showed the importance of setting the initial value of this prior being equal to 1/K for improved accuracy while analyzing unbalanced populations (as in our case, we had RR, RL, but not LL individuals). STRUCTURE analysis of the phased dataset was run with the same settings.

PCA on diploid and phased SNP datasets was performed with the Pegas (Paradis 2010), *radiator*^53^, and *adegenet*^57^ R packages. To explore relationships between phased haplotypes, we constructed SNP dendrogram (additive branch length) with the R package SNPRelate v1.16.0^58^ using a set of 2219 SNPs, i.e., without filtering on presence/absence of loci to account for null allele variation between the samples (Supplementary Figure S4).

To investigate the traces of possible recombination within the germline of the RL males, we reconstructed the sex-wise linkage maps using diploid genotypes of the parents and progeny. In sexual females, which recombine, loci were expected to distribute along with the linkage groups; however, in hemiclonal males, the distance between markers was expected to be 0 cM. We reconstructed linkage maps using programs Lep-Map3^59^ and MSTmap^60,61^. In order to assess coancestry and genomic variation among phased R-sexual and [R]-/[L]-clonal haplotypes, we used the fineRADstructure pipeline^62^ of the fineSTRUCTURE Markov chain Monte Carlo (MCMC) clustering algorithm^63^. This method takes into account linkage information between loci and generates a coancestry matrix accounting for the closest neighboring haplotype, the data undergoes MCMC sampling, and ultimately closely related genetic clusters are inferred. Phased data in the form of a VCF file were converted into fineRADstructure input with a genomic converter module of *radiator* v 0.0.18 R package^53^. Since our dataset was mapped on a draft genome assembly that does not reflect physical linkage to a large extent, we corrected for linkage disequilibrium with the sampleLD R-script following the recommendations of fineRADstructure developers.

### Ethical approval

All experimental protocols were approved by the Federal Commission for Animal Experiments (FCAE) and by the Standing committee on animal health under the Federal Food Safety and Veterinary Office FSVO, Switzerland, under permit nos 119/2013: TV 5113 and TH 103. We declare that all other manipulations with animals were performed in accordance with relevant guidelines and regulations. The study was carried out in compliance with the ARRIVE guidelines.

## Supplementary Information

Supplementary Information.

## Data Availability

All the microsatellite data supporting our results and conclusions, along with sufficient details, are included in the manuscript and Supplementary files. The initial *P. lessonae* genome assembly is available at https://blast.naturkundemuseum.berlin/frog/. ddRADseq libraries are accessible under the BioProject ID PRJNA662869.
