# Supplementary material for: Capture and return of sexual genomes by hybridogenetic frogs provide clonal genome enrichment in a sexual species
Source: Sci Rep. 2021 Jan 15;11:1633. doi: 10.1038/s41598-021-81240-5 (PMC7810977; doi:10.1038/s41598-021-81240-5)
Supplement: Supplementary file 1 — Supplementary Information. [file 41598_2021_81240_MOESM1_ESM.docx]

**Supplementary files**

Title of the manuscript:

**Capture and return of sexual genomes by hybridogenetic frogs provide clonal genome enrichment in a sexual species**

Author list:

Marie Doležálková-Kaštánková, Glib Mazepa, Daniel L. Jeffries, Nicolas Perrin, Marcela Plötner, Jörg Plötner, Gaston-Denis Guex, Peter Mikulíček, Albert J. Poustka, Jose Grau and Lukáš Choleva

Supplementary Table S1: Crossing design scheme with a number of metamorphosed juveniles (in the brackets). M = male, F = female, RR = *P. ridibundus*, RL = *P. esculentus*.

| **M/F** | **F1 RR** | **F2 RR** | **F3 RR** | **F4 RR** | **F5 RR** | **F6 RR** | **F7 RR** | **F8 RR** | **F9 RR** |
| --- | --- | --- | --- | --- | --- | --- | --- | --- | --- |
| **K RR** | 10-2013 | 16-2013 | 47-2013 | 48-2013 | 50-2013 | 51-2013 | 58-2013 | 67-2013 | 72-2013 |
| **M1 RL** | 25-2013 (137) | 32-2013 (32) |  |  |  |  |  |  |  |
| **M2 RL** | 26-2013 (17) | 31-2013 (0) |  |  |  |  |  |  |  |
| **M3 RL** | 27-2013 (29) | 30-2013 (76) |  |  |  |  |  |  |  |
| **M4 RL** | 28-2013 (0) | 29-2013 (0) |  |  |  |  |  |  |  |
| **M5 RL** |  |  | 39-2013 (23) | 49-2013 (2) | 52-2013 (0) |  |  |  |  |
| **M6 RL** |  |  | 40-2013 (0) |  |  | 44-2013 (0) |  |  |  |
| **M7 RL** |  |  | 41-2013 (74) |  |  | 45-2013 (0) |  |  |  |
| **M8 RL** |  |  | 42-2013 (66) |  |  | 46-2013 (0) |  |  |  |
| **M9 RL** |  |  |  |  |  |  | 53-2013 (2) | 62-2013 (0) |  |
| **M10 RL** |  |  |  |  |  |  | 54-2013 (2) | 63-2013 (3) |  |
| **M11 RL** |  |  |  |  |  |  | 55-2013 (0) | 64-2013 (0) |  |
| **M12 RL** |  |  |  |  |  |  | 56-2013 (0) | 65-2013 (1) |  |
| **M13 RL** |  |  |  |  |  |  | 57-2013 (3) | 66-2013 (33) |  |
| **M14 RL** |  |  |  |  |  |  | 59-2013 (3) | 68-2013 (0) | 69-2013 (0) |
| **M15 RL** |  |  |  |  |  |  | 60-2013 (20) |  | 70-2013 (0) |
| **M16 RL** |  |  |  |  |  |  | 61-2013 (0) |  | 71-2013 (0) |

Supplementary Table S2. A list of adult *Pelpphylax* water frogs used for crossing experiments. nuDNA=nuclear DNA.

| **Male ID** | **Individual Code** | **nuDNA** | **Origin** | **Female ID** | **Individual Code** | **nuDNA** | **Origin** |
| --- | --- | --- | --- | --- | --- | --- | --- |
| **C** | 13CZ3WF21M | RR | Košatka | **F1** | 13CZ1WF3F | RR | Cítov |
| **M1** | 13CZ3WF28M | RL | Košatka | **F2** | 13CZ3WF10F | RR | Košatka |
| **M2** | 13CZ3WF29M | RL | Košatka | **F3** | 13CZ3WF11F | RR | Košatka |
| **M3** | 13CZ3WF30M | RL | Košatka | **F4** | 13CZ2WF1F | RR | Liběchov |
| **M4** | 13CZ3WF31M | RL | Košatka | **F5** | 13CZ4WF1F | RR | Dolní Benešov |
| **M5** | 13CZ3WF32M | RL | Košatka | **F6** | 13CZ3WF3F | RR | Košatka |
| **M6** | 13CZ3WF33M | RL | Košatka | **F7** | 13CZ1WF4F | RR | Cítov |
| **M7** | 13CZ3WF35M | RL | Košatka | **F8** | 54F3 (2012) | RR | Cítov |
| **M8** | 13CZ3WF36M | RL | Košatka | **F9** | 13CZ3WF2F | RR | Košatka |
| **M9** | 13CZ3WF38M | RL | Košatka |  |  |  |  |
| **M10** | 13CZ3WF39M | RL | Košatka |  |  |  |  |
| **M11** | 13CZ3WF40M | RL | Košatka |  |  |  |  |
| **M12** | 13CZ3WF42M | RL | Košatka |  |  |  |  |
| **M13** | 13CZ3WF43M | RL | Košatka |  |  |  |  |
| **M14** | 13CZ3WF44M | RL | Košatka |  |  |  |  |
| **M15** | 13CZ3WF45M | RL | Košatka |  |  |  |  |
| **M16** | 13CZ5WF1M | RL | Albrechtičky |  |  |  |  |

Supplementary Table S3. Microsatellite data (Multiplex 1 and 2) of 238 individuals from 17 families. Each groups starts with mother and father followed by their progeny. R-specific alleles are in red, L-specific alleles are in green. F = female, M = male, RR = *Pelophylax ridibundus*, RL = *P. esculentus*, Gen = genotype.

| Multiplex 1 |  |  | | Microsatellite loci | | | | | | | | | | | | | | | | | | | | | | | |  |  |  |  |  |  |  |  |  |
| --- | --- | --- | --- | --- | --- | --- | --- | --- | --- | --- | --- | --- | --- | --- | --- | --- | --- | --- | --- | --- | --- | --- | --- | --- | --- | --- | --- | --- | --- | --- | --- | --- | --- | --- | --- | --- |
| **Individual ID** | **Sex** | **Gen** | | **RICA2a34** | | | **RICA5** | | | **Rrid013A** | | | | **Ga1a19** | | | **RICA18** | | | **RICA1b5** | | **Res14** | | | **Res20** | | |  |  |  |  |  |  |  |  |  |
| 13CZ3WF28M | M | RL | | 0 | 144 | | 0 | | 262 | 293 | | 301 | | 207 | 197 | | 0 | 188 | | 136 | 120 | 150 | | 140 | 0 | | 124 |  |  |  |  |  |  |  |  |  |
| 13CZ1WF3F | F | RR | | 0 | 0 | | 0 | | 0 | 287 | | 287 | | 207 | 207 | | 0 | 0 | | 136 | 136 | 146 | | 146 | 0 | | 0 |  |  |  |  |  |  |  |  |  |
| 25_2013JUV1 | M | RL | | 0 | 144 | | 0 | | 262 | 287 | | 301 | | 207 | 197 | | 0 | 188 | | 136 | 120 | 0 | | 140 | 0 | | 124 |  |  |  |  |  |  |  |  |  |
| 25_2013JUV10 | M | RL | | 0 | 144 | | 0 | | 262 | 287 | | 301 | | 207 | 197 | | 0 | 188 | | 136 | 120 | 146 | | 140 | 0 | | 124 |  |  |  |  |  |  |  |  |  |
| 25_2013JUV11 | M | RL | | 0 | 144 | | 0 | | 262 | 287 | | 301 | | 207 | 197 | | 0 | 188 | | 136 | 120 | 146 | | 140 | 0 | | 124 |  |  |  |  |  |  |  |  |  |
| 25_2013JUV12 | M | RL | | 0 | 144 | | 0 | | 262 | 287 | | 301 | | 207 | 197 | | 0 | 188 | | 136 | 120 | 0 | | 140 | 0 | | 0 |  |  |  |  |  |  |  |  |  |
| 25_2013JUV13 | M | RL | | 0 | 144 | | 0 | | 262 | 287 | | 301 | | 207 | 197 | | 0 | 188 | | 136 | 120 | 0 | | 140 | 0 | | 0 |  |  |  |  |  |  |  |  |  |
| 25_2013JUV14 | M | RL | | 0 | 144 | | 0 | | 262 | 287 | | 301 | | 207 | 197 | | 0 | 188 | | 136 | 120 | 0 | | 140 | 0 | | 124 |  |  |  |  |  |  |  |  |  |
| 25_2013JUV15 | M | RL | | 0 | 144 | | 0 | | 262 | 287 | | 301 | | 207 | 197 | | 0 | 188 | | 136 | 120 | 0 | | 140 | 0 | | 0 |  |  |  |  |  |  |  |  |  |
| 25_2013JUV16 | M | RL | | 0 | 144 | | 0 | | 262 | 287 | | 301 | | 207 | 197 | | 0 | 188 | | 136 | 120 | 0 | | 140 | 0 | | 0 |  |  |  |  |  |  |  |  |  |
| 25_2013JUV17 | M | RL | | 0 | 144 | | 0 | | 262 | 287 | | 301 | | 207 | 197 | | 0 | 188 | | 136 | 120 | 146 | | 140 | 0 | | 0 |  |  |  |  |  |  |  |  |  |
| 25_2013JUV18 | M | RL | | 0 | 144 | | 0 | | 262 | 287 | | 301 | | 207 | 197 | | 0 | 188 | | 136 | 120 | 146 | | 140 | 0 | | 0 |  |  |  |  |  |  |  |  |  |
| 25_2013JUV19 | M | RL | | 0 | 144 | | 0 | | 262 | 287 | | 301 | | 207 | 197 | | 0 | 188 | | 136 | 120 | 0 | | 140 | 0 | | 0 |  |  |  |  |  |  |  |  |  |
| 25_2013JUV2 | M | RL | | 0 | 144 | | 0 | | 262 | 287 | | 301 | | 207 | 197 | | 0 | 188 | | 136 | 120 | 146 | | 140 | 0 | | 124 |  |  |  |  |  |  |  |  |  |
| 25_2013JUV20 | M | RL | | 0 | 144 | | 0 | | 262 | 287 | | 301 | | 207 | 197 | | 0 | 188 | | 136 | 120 | 0 | | 140 | 0 | | 0 |  |  |  |  |  |  |  |  |  |
| 25_2013JUV3 | M | RL | | 0 | 144 | | 0 | | 262 | 287 | | 301 | | 207 | 197 | | 0 | 188 | | 136 | 120 | 0 | | 140 | 0 | | 124 |  |  |  |  |  |  |  |  |  |
| 25_2013JUV4 | M | RL | | 0 | 144 | | 0 | | 262 | 287 | | 301 | | 207 | 197 | | 0 | 188 | | 136 | 120 | 146 | | 140 | 0 | | 124 |  |  |  |  |  |  |  |  |  |
| 25_2013JUV5 | M | RL | | 0 | 144 | | 0 | | 262 | 287 | | 301 | | 207 | 197 | | 0 | 188 | | 136 | 120 | 146 | | 140 | 0 | | 124 |  |  |  |  |  |  |  |  |  |
| 25_2013JUV6 | M | RL | | 0 | 144 | | 0 | | 262 | 287 | | 301 | | 207 | 197 | | 0 | 188 | | 136 | 120 | 146 | | 140 | 0 | | 124 |  |  |  |  |  |  |  |  |  |
| 25_2013JUV7 | M | RL | | 0 | 144 | | 0 | | 262 | 287 | | 301 | | 207 | 197 | | 0 | 188 | | 136 | 120 | 146 | | 140 | 0 | | 124 |  |  |  |  |  |  |  |  |  |
| 25_2013JUV8 | M | RL | | 0 | 144 | | 0 | | 262 | 287 | | 301 | | 207 | 197 | | 0 | 188 | | 136 | 120 | 146 | | 140 | 0 | | 124 |  |  |  |  |  |  |  |  |  |
| 25_2013JUV9 | M | RL | | 0 | 144 | | 0 | | 262 | 287 | | 301 | | 207 | 197 | | 0 | 188 | | 136 | 120 | 146 | | 140 | 0 | | 124 |  |  |  |  |  |  |  |  |  |
| 13CZ5WF1M | M | RL | | 0 | 144 | | 0 | | 262 | 287 | | 301 | | 207 | 197 | | 0 | 188 | | 136 | 120 | 146 | | 140 | 0 | | 124 |  |  |  |  |  |  |  |  |  |
| 13CZ1WF3F | F | RR | | 0 | 0 | | 0 | | 0 | 287 | | 287 | | 207 | 207 | | 0 | 0 | | 136 | 136 | 146 | | 146 | 0 | | 0 |  |  |  |  |  |  |  |  |  |
| 26_2013JUV1 | M | RL | | 0 | 144 | | 0 | | 262 | 287 | | 301 | | 207 | 197 | | 0 | 188 | | 136 | 120 | 0 | | 140 | 0 | | 124 |  |  |  |  |  |  |  |  |  |
| 26_2013JUV2 | M | RL | | 0 | 144 | | 0 | | 262 | 287 | | 301 | | 207 | 197 | | 0 | 188 | | 136 | 120 | 0 | | 140 | 0 | | 124 |  |  |  |  |  |  |  |  |  |
| 26_2013JUV3 | M | RL | | 0 | 144 | | 0 | | 262 | 287 | | 301 | | 207 | 197 | | 0 | 188 | | 136 | 120 | 0 | | 140 | 0 | | 124 |  |  |  |  |  |  |  |  |  |
| 26_2013JUV10 | M | RL | | 0 | 144 | | 0 | | 262 | 287 | | 301 | | 207 | 197 | | 0 | 188 | | 136 | 120 | 0 | | 140 | 0 | | 124 |  |  |  |  |  |  |  |  |  |
| 26_2013JUV11 | M | RL | | 0 | 144 | | 0 | | 262 | 287 | | 301 | | 207 | 197 | | 0 | 188 | | 136 | 120 | 0 | | 140 | 0 | | 0 |  |  |  |  |  |  |  |  |  |
| 26_2013JUV12 | M | RL | | 0 | 144 | | 0 | | 262 | 287 | | 301 | | 207 | 197 | | 0 | 188 | | 136 | 120 | 146 | | 140 | 0 | | 0 |  |  |  |  |  |  |  |  |  |
| 26_2013JUV13 | M | RL | | 0 | 144 | | 0 | | 262 | 287 | | 301 | | 207 | 197 | | 0 | 188 | | 136 | 120 | 0 | | 140 | 0 | | 0 |  |  |  |  |  |  |  |  |  |
| 26_2013JUV14 | M | RL | | 0 | 144 | | 0 | | 262 | 287 | | 301 | | 207 | 197 | | 0 | 188 | | 136 | 120 | 146 | | 140 | 0 | | 0 |  |  |  |  |  |  |  |  |  |
| 26_2013JUV15 | M | RL | | 0 | 144 | | 0 | | 262 | 287 | | 301 | | 207 | 197 | | 0 | 188 | | 136 | 120 | 0 | | 140 | 0 | | 0 |  |  |  |  |  |  |  |  |  |
| 26_2013JUV16 | M | RL | | 0 | 144 | | 0 | | 262 | 287 | | 301 | | 207 | 197 | | 0 | 188 | | 136 | 120 | 146 | | 140 | 0 | | 0 |  |  |  |  |  |  |  |  |  |
| 26_2013JUV4 | M | RL | | 0 | 144 | | 0 | | 262 | 287 | | 301 | | 207 | 197 | | 0 | 188 | | 136 | 120 | 0 | | 140 | 0 | | 124 |  |  |  |  |  |  |  |  |  |
| 26_2013JUV5 | M | RL | | 0 | 144 | | 0 | | 262 | 287 | | 301 | | 207 | 197 | | 0 | 188 | | 136 | 120 | 146 | | 140 | 0 | | 124 |  |  |  |  |  |  |  |  |  |
| 26_2013JUV6 | M | RL | | 0 | 144 | | 0 | | 262 | 287 | | 301 | | 207 | 197 | | 0 | 188 | | 136 | 120 | 146 | | 140 | 0 | | 124 |  |  |  |  |  |  |  |  |  |
| 26_2013JUV7 | M | RL | | 0 | 144 | | 0 | | 262 | 287 | | 301 | | 207 | 197 | | 0 | 188 | | 136 | 120 | 0 | | 140 | 0 | | 124 |  |  |  |  |  |  |  |  |  |
| 26_2013JUV8 | M | RL | | 0 | 144 | | 0 | | 262 | 287 | | 301 | | 207 | 197 | | 0 | 188 | | 136 | 120 | 0 | | 140 | 0 | | 124 |  |  |  |  |  |  |  |  |  |
| 26_2013JUV9 | M | RL | | 0 | 144 | | 0 | | 262 | 287 | | 301 | | 207 | 197 | | 0 | 188 | | 136 | 120 | 0 | | 140 | 0 | | 124 |  |  |  |  |  |  |  |  |  |
| 13CZ3WF40M | M | RL | | 0 | 144 | | 0 | | 262 | 293 | | 301 | | 245 | 197 | | 0 | 188 | | 136 | 120 | 146 | | 140 | 0 | | 124 |  |  |  |  |  |  |  |  |  |
| 13CZ1WF3F | F | RR | | 0 | 0 | | 0 | | 0 | 287 | | 287 | | 207 | 207 | | 0 | 0 | | 136 | 136 | 146 | | 146 | 0 | | 0 |  |  |  |  |  |  |  |  |  |
| 27_2013JUV10 | M | RL | | 0 | 144 | | 0 | | 262 | 287 | | 301 | | 0 | 0 | | 0 | 188 | | 136 | 120 | 0 | | 140 | 0 | | 124 |  |  |  |  |  |  |  |  |  |
| 27_2013JUV11 | F | RR | | 0 | 0 | | 0 | | 0 | 287 | | 293 | | 207 | 245 | | 0 | 0 | | 136 | 136 | 146 | | 146 | 0 | | 0 |  |  |  |  |  |  |  |  |  |
| 27_2013JUV12 | F | RR | | 0 | 0 | | 0 | | 0 | 287 | | 293 | | 207 | 245 | | 0 | 0 | | 136 | 136 | 146 | | 146 | 0 | | 0 |  |  |  |  |  |  |  |  |  |
| 27_2013JUV13 | F | RR | | 0 | 0 | | 0 | | 0 | 287 | | 293 | | 207 | 245 | | 0 | 0 | | 136 | 136 | 146 | | 146 | 0 | | 0 |  |  |  |  |  |  |  |  |  |
| 27_2013JUV14 | F | RR | | 0 | 0 | | 0 | | 0 | 287 | | 293 | | 207 | 245 | | 0 | 0 | | 136 | 136 | 146 | | 146 | 0 | | 0 |  |  |  |  |  |  |  |  |  |
| 27_2013JUV15 | F | RR | | 0 | 0 | | 0 | | 0 | 287 | | 293 | | 207 | 245 | | 0 | 0 | | 136 | 136 | 146 | | 146 | 0 | | 0 |  |  |  |  |  |  |  |  |  |
| 27_2013JUV16 | F | RR | | 0 | 0 | | 0 | | 0 | 287 | | 293 | | 207 | 245 | | 0 | 0 | | 136 | 136 | 146 | | 146 | 0 | | 0 |  |  |  |  |  |  |  |  |  |
| 27_2013JUV17 | M | RL | | 0 | 144 | | 0 | | 262 | 287 | | 301 | | 207 | 197 | | 0 | 188 | | 136 | 120 | 146 | | 140 | 0 | | 0 |  |  |  |  |  |  |  |  |  |
| 27_2013JUV18 | F | RR | | 0 | 0 | | 0 | | 0 | 287 | | 293 | | 207 | 245 | | 0 | 0 | | 136 | 136 | 146 | | 146 | 0 | | 0 |  |  |  |  |  |  |  |  |  |
| 27_2013JUV1 | M | RL | | 0 | 144 | | 0 | | 262 | 287 | | 301 | | 207 | 197 | | 0 | 188 | | 136 | 120 | 0 | | 140 | 0 | | 124 |  |  |  |  |  |  |  |  |  |
| 27_2013JUV21 | F | RR | | 0 | 0 | | 0 | | 0 | 287 | | 293 | | 207 | 245 | | 0 | 0 | | 136 | 136 | 146 | | 146 | 0 | | 0 |  |  |  |  |  |  |  |  |  |
| 27_2013JUV22 | F | RR | | 0 | 0 | | 0 | | 0 | 287 | | 293 | | 207 | 245 | | 0 | 0 | | 136 | 136 | 146 | | 146 | 0 | | 0 |  |  |  |  |  |  |  |  |  |
| 27_2013JUV23 | F | RR | | 0 | 0 | | 0 | | 0 | 287 | | 293 | | 207 | 245 | | 0 | 0 | | 136 | 136 | 146 | | 146 | 0 | | 0 |  |  |  |  |  |  |  |  |  |
| 27_2013JUV24 | F | RR | | 0 | 0 | | 0 | | 0 | 287 | | 293 | | 207 | 245 | | 0 | 0 | | 136 | 136 | 146 | | 146 | 0 | | 0 |  |  |  |  |  |  |  |  |  |
| 27_2013JUV25 | F | RR | | 0 | 0 | | 0 | | 0 | 287 | | 293 | | 207 | 245 | | 0 | 0 | | 136 | 136 | 146 | | 146 | 0 | | 0 |  |  |  |  |  |  |  |  |  |
| 27_2013JUV26 | F | RR | | 0 | 0 | | 0 | | 0 | 287 | | 293 | | 207 | 245 | | 0 | 0 | | 136 | 136 | 146 | | 146 | 0 | | 0 |  |  |  |  |  |  |  |  |  |
| 27_2013JUV27 | F | RR | | 0 | 0 | | 0 | | 0 | 287 | | 293 | | 207 | 245 | | 0 | 0 | | 136 | 136 | 146 | | 146 | 0 | | 0 |  |  |  |  |  |  |  |  |  |
| 27_2013JUV28 | F | RR | | 0 | 0 | | 0 | | 0 | 287 | | 293 | | 207 | 245 | | 0 | 0 | | 136 | 136 | 146 | | 146 | 0 | | 0 |  |  |  |  |  |  |  |  |  |
| 27_2013JUV2 | M | RL | | 0 | 144 | | 0 | | 262 | 287 | | 301 | | 207 | 197 | | 0 | 188 | | 136 | 120 | 0 | | 140 | 0 | | 124 |  |  |  |  |  |  |  |  |  |
| 27_2013JUV3 | M | RL | | 0 | 144 | | 0 | | 262 | 287 | | 301 | | 207 | 197 | | 0 | 188 | | 136 | 120 | 0 | | 140 | 0 | | 124 |  |  |  |  |  |  |  |  |  |
| 27_2013JUV4 | M | RL | | 0 | 144 | | 0 | | 262 | 287 | | 301 | | 207 | 197 | | 0 | 188 | | 136 | 120 | 0 | | 140 | 0 | | 124 |  |  |  |  |  |  |  |  |  |
| 27_2013JUV5 | M | RL | | 0 | 0 | | 0 | | 0 | 0 | | 0 | | 207 | 197 | | 0 | 188 | | 0 | 0 | 0 | | 0 | 0 | | 124 |  |  |  |  |  |  |  |  |  |
| 27_2013JUV6 | M | RL | | 0 | 144 | | 0 | | 262 | 287 | | 301 | | 207 | 197 | | 0 | 188 | | 136 | 120 | 146 | | 140 | 0 | | 124 |  |  |  |  |  |  |  |  |  |
| 27_2013JUV7 | M | RL | | 0 | 144 | | 0 | | 262 | 287 | | 301 | | 207 | 197 | | 0 | 188 | | 136 | 120 | 0 | | 140 | 0 | | 124 |  |  |  |  |  |  |  |  |  |
| 27_2013JUV8 | M | RL | | 0 | 144 | | 0 | | 262 | 287 | | 301 | | 207 | 197 | | 0 | 188 | | 136 | 120 | 146 | | 140 | 0 | | 124 |  |  |  |  |  |  |  |  |  |
| 27_2013JUV9 | M | RL | | 0 | 144 | | 0 | | 262 | 287 | | 301 | | 207 | 197 | | 0 | 188 | | 136 | 120 | 146 | | 140 | 0 | | 124 |  |  |  |  |  |  |  |  |  |
| 13CZ3WF40M | M | RL | | 0 | 144 | | 0 | | 262 | 293 | | 301 | | 245 | 197 | | 0 | 188 | | 136 | 120 | 146 | | 140 | 0 | | 124 |  |  |  |  |  |  |  |  |  |
| 13CZ3WF10F | F | RR | | 0 | 0 | | 0 | | 0 | 287 | | 293 | | 207 | 224 | | 0 | 0 | | 136 | 136 | 150 | | 150 | 0 | | 0 |  |  |  |  |  |  |  |  |  |
| 30_2013JUV1 | M | RL | | 0 | 144 | | 0 | | 262 | 293 | | 301 | | 0 | 0 | | 0 | 188 | | 136 | 120 | 150 | | 140 | 0 | | 124 |  |  |  |  |  |  |  |  |  |
| 30_2013JUV10 | M | RL | | 0 | 144 | | 0 | | 262 | 293 | | 301 | | 0 | 0 | | 0 | 188 | | 136 | 120 | 150 | | 140 | 0 | | 124 |  |  |  |  |  |  |  |  |  |
| 30_2013JUV10B | M | RL | | 0 | 144 | | 0 | | 262 | 293 | | 301 | | 0 | 0 | | 0 | 188 | | 136 | 120 | 150 | | 140 | 0 | | 124 |  |  |  |  |  |  |  |  |  |
| 30_2013JUV11 | F | RR | | 0 | 0 | | 0 | | 0 | 0 | | 0 | | 0 | 0 | | 0 | 0 | | 0 | 0 | 0 | | 0 | 0 | | 0 |  |  |  |  |  |  |  |  |  |
| 30_2013JUV11B | M | RL | | 0 | 144 | | 0 | | 262 | 293 | | 301 | | 0 | 0 | | 0 | 188 | | 136 | 120 | 150 | | 140 | 0 | | 124 |  |  |  |  |  |  |  |  |  |
| 30_2013JUV12B | M | RL | | 0 | 0 | | 0 | | 0 | 0 | | 0 | | 0 | 0 | | 0 | 0 | | 0 | 0 | 0 | | 0 | 0 | | 0 |  |  |  |  |  |  |  |  |  |
| 30_2013JUV13 | F | RR | | 0 | 0 | | 0 | | 0 | 287 | | 293 | | 0 | 0 | | 0 | 0 | | 136 | 136 | 146 | | 150 | 0 | | 0 |  |  |  |  |  |  |  |  |  |
| 30_2013JUV13B | M | RL | | 0 | 144 | | 0 | | 262 | 293 | | 301 | | 0 | 0 | | 0 | 0 | | 136 | 120 | 150 | | 140 | 0 | | 124 |  |  |  |  |  |  |  |  |  |
| 30_2013JUV14 | M | RL | | 0 | 144 | | 0 | | 262 | 287 | | 301 | | 0 | 0 | | 0 | 188 | | 136 | 120 | 150 | | 140 | 0 | | 124 |  |  |  |  |  |  |  |  |  |
| 30_2013JUV14B | M | RL | | 0 | 144 | | 0 | | 262 | 293 | | 301 | | 0 | 0 | | 0 | 188 | | 136 | 120 | 150 | | 140 | 0 | | 124 |  |  |  |  |  |  |  |  |  |
| 30_2013JUV17 | F | RR | | 0 | 0 | | 0 | | 0 | 287 | | 293 | | 0 | 0 | | 0 | 0 | | 136 | 136 | 146 | | 150 | 0 | | 0 |  |  |  |  |  |  |  |  |  |
| 30_2013JUV18 | F | RR | | 0 | 0 | | 0 | | 0 | 287 | | 293 | | 0 | 0 | | 0 | 0 | | 136 | 136 | 146 | | 150 | 0 | | 0 |  |  |  |  |  |  |  |  |  |
| 30_2013JUV19 | F | RR | | 0 | 0 | | 0 | | 0 | 293 | | 293 | | 0 | 0 | | 0 | 0 | | 136 | 136 | 146 | | 150 | 0 | | 0 |  |  |  |  |  |  |  |  |  |
| 30_2013JUV1B | M | RL | | 0 | 144 | | 0 | | 262 | 293 | | 301 | | 0 | 0 | | 0 | 188 | | 136 | 120 | 150 | | 140 | 0 | | 124 |  |  |  |  |  |  |  |  |  |
| 30_2013JUV2 | F | RR | | 0 | 0 | | 0 | | 0 | 293 | | 293 | | 0 | 0 | | 0 | 0 | | 136 | 136 | 146 | | 150 | 0 | | 0 |  |  |  |  |  |  |  |  |  |
| 30_2013JUV20 | F | RR | | 0 | 0 | | 0 | | 0 | 293 | | 293 | | 0 | 0 | | 0 | 0 | | 0 | 0 | 146 | | 150 | 0 | | 0 |  |  |  |  |  |  |  |  |  |
| 30_2013JUV21 | F | RR | | 0 | 0 | | 0 | | 0 | 287 | | 293 | | 0 | 0 | | 0 | 0 | | 136 | 136 | 146 | | 150 | 0 | | 0 |  |  |  |  |  |  |  |  |  |
| 30_2013JUV22 | M | RL | | 0 | 144 | | 0 | | 262 | 293 | | 301 | | 0 | 0 | | 0 | 0 | | 0 | 0 | 150 | | 140 | 0 | | 124 |  |  |  |  |  |  |  |  |  |
| 30_2013JUV23 | F | RR | | 0 | 0 | | 0 | | 0 | 293 | | 293 | | 0 | 0 | | 0 | 0 | | 136 | 136 | 146 | | 150 | 0 | | 0 |  |  |  |  |  |  |  |  |  |
| 30_2013JUV24 | F | RR | | 0 | 0 | | 0 | | 0 | 293 | | 293 | | 0 | 0 | | 0 | 0 | | 136 | 136 | 146 | | 150 | 0 | | 0 |  |  |  |  |  |  |  |  |  |
| 30_2013JUV25 | F | RR | | 0 | 0 | | 0 | | 0 | 293 | | 293 | | 0 | 0 | | 0 | 0 | | 136 | 136 | 146 | | 150 | 0 | | 0 |  |  |  |  |  |  |  |  |  |
| 30_2013JUV2B | M | RL | | 0 | 144 | | 0 | | 262 | 293 | | 301 | | 0 | 0 | | 0 | 188 | | 136 | 120 | 150 | | 140 | 0 | | 124 |  |  |  |  |  |  |  |  |  |
| 30_2013JUV3 | F | RR | | 0 | 0 | | 0 | | 0 | 293 | | 293 | | 0 | 0 | | 0 | 0 | | 136 | 136 | 146 | | 150 | 0 | | 0 |  |  |  |  |  |  |  |  |  |
| 30_2013JUV32 | M | RL | | 0 | 144 | | 0 | | 262 | 287 | | 301 | | 0 | 0 | | 0 | 188 | | 136 | 120 | 150 | | 140 | 0 | | 124 |  |  |  |  |  |  |  |  |  |
| 30_2013JUV33 | M | RL | | 0 | 0 | | 0 | | 0 | 0 | | 0 | | 0 | 0 | | 0 | 0 | | 0 | 0 | 0 | | 0 | 0 | | 0 |  |  |  |  |  |  |  |  |  |
| 30_2013JUV38 | F | RR | | 0 | 0 | | 0 | | 0 | 293 | | 293 | | 0 | 0 | | 0 | 0 | | 136 | 136 | 0 | | 146 | 0 | | 0 |  |  |  |  |  |  |  |  |  |
| 30_2013JUV3B | M | RL | | 0 | 144 | | 0 | | 262 | 287 | | 301 | | 0 | 0 | | 0 | 188 | | 136 | 120 | 150 | | 140 | 0 | | 124 |  |  |  |  |  |  |  |  |  |
| 30_2013JUV4 | F | RR | | 0 | 0 | | 0 | | 0 | 293 | | 293 | | 0 | 0 | | 0 | 0 | | 136 | 136 | 146 | | 150 | 0 | | 0 |  |  |  |  |  |  |  |  |  |
| 30_2013JUV4B | M | RL | | 0 | 144 | | 0 | | 262 | 287 | | 301 | | 0 | 0 | | 0 | 188 | | 136 | 120 | 150 | | 140 | 0 | | 124 |  |  |  |  |  |  |  |  |  |
| 30_2013JUV5 | M | RL | | 0 | 144 | | 0 | | 262 | 287 | | 301 | | 0 | 0 | | 0 | 188 | | 136 | 120 | 150 | | 140 | 0 | | 124 |  |  |  |  |  |  |  |  |  |
| 30_2013JUV5B | M | RL | | 0 | 144 | | 0 | | 262 | 287 | | 301 | | 0 | 0 | | 0 | 188 | | 136 | 120 | 150 | | 140 | 0 | | 124 |  |  |  |  |  |  |  |  |  |
| 30_2013JUV6 | M | RL | | 0 | 144 | | 0 | | 262 | 293 | | 301 | | 0 | 0 | | 0 | 188 | | 136 | 120 | 150 | | 140 | 0 | | 124 |  |  |  |  |  |  |  |  |  |
| 30_2013JUV6B | M | RL | | 0 | 144 | | 0 | | 262 | 293 | | 301 | | 0 | 0 | | 0 | 188 | | 136 | 120 | 150 | | 140 | 0 | | 124 |  |  |  |  |  |  |  |  |  |
| 30_2013JUV7 | F | RR | | 0 | 0 | | 0 | | 0 | 287 | | 293 | | 0 | 0 | | 0 | 0 | | 136 | 136 | 146 | | 150 | 0 | | 0 |  |  |  |  |  |  |  |  |  |
| 30_2013JUV7B | M | RL | | 0 | 144 | | 0 | | 262 | 287 | | 301 | | 0 | 0 | | 0 | 188 | | 136 | 120 | 150 | | 140 | 0 | | 124 |  |  |  |  |  |  |  |  |  |
| 30_2013JUV8 | F | RR | | 0 | 0 | | 0 | | 0 | 293 | | 293 | | 0 | 0 | | 0 | 0 | | 136 | 136 | 146 | | 150 | 0 | | 0 |  |  |  |  |  |  |  |  |  |
| 30_2013JUV8B | M | RL | | 0 | 144 | | 0 | | 262 | 287 | | 301 | | 0 | 0 | | 0 | 188 | | 136 | 120 | 150 | | 140 | 0 | | 124 |  |  |  |  |  |  |  |  |  |
| 30_2013JUV9B | M | RL | | 0 | 144 | | 0 | | 262 | 293 | | 301 | | 0 | 0 | | 0 | 188 | | 136 | 120 | 150 | | 140 | 0 | | 124 |  |  |  |  |  |  |  |  |  |
| 30_2013JUV12 | F | RR | | 0 | 0 | | 0 | | 0 | 293 | | 293 | | 207 | 245 | | 0 | 0 | | 136 | 136 | 146 | | 150 | 0 | | 0 |  |  |  |  |  |  |  |  |  |
| 30_2013JUV15 | F | RR | | 0 | 0 | | 0 | | 0 | 293 | | 293 | | 224 | 245 | | 0 | 0 | | 136 | 136 | 146 | | 150 | 0 | | 0 |  |  |  |  |  |  |  |  |  |
| 30_2013JUV16 | F | RR | | 0 | 0 | | 0 | | 0 | 293 | | 293 | | 224 | 245 | | 0 | 0 | | 136 | 136 | 146 | | 150 | 0 | | 0 |  |  |  |  |  |  |  |  |  |
| 30_2013JUV9 | F | RR | | 0 | 0 | | 0 | | 0 | 293 | | 293 | | 224 | 245 | | 0 | 0 | | 136 | 136 | 146 | | 150 | 0 | | 0 |  |  |  |  |  |  |  |  |  |
| 13CZ3WF28M | M | RL | | 0 | 144 | | 0 | | 262 | 293 | | 301 | | 207 | 197 | | 0 | 188 | | 136 | 120 | 150 | | 140 | 0 | | 124 |  |  |  |  |  |  |  |  |  |
| 13CZ3WF10F | F | RR | | 0 | 0 | | 0 | | 0 | 287 | | 293 | | 207 | 224 | | 0 | 0 | | 136 | 136 | 150 | | 150 | 0 | | 0 |  |  |  |  |  |  |  |  |  |
| 32_2013JUV1B | M | RL | | 0 | 144 | | 0 | | 262 | 293 | | 301 | | 224 | 197 | | 0 | 188 | | 136 | 120 | 150 | | 140 | 0 | | 0 |  |  |  |  |  |  |  |  |  |
| 32_2013JUV1 | M | RL | | 0 | 144 | | 0 | | 262 | 287 | | 301 | | 224 | 197 | | 0 | 188 | | 136 | 120 | 150 | | 140 | 0 | | 0 |  |  |  |  |  |  |  |  |  |
| 32_2013JUV2 | M | RL | | 0 | 144 | | 0 | | 262 | 293 | | 301 | | 207 | 197 | | 0 | 188 | | 136 | 120 | 150 | | 140 | 0 | | 0 |  |  |  |  |  |  |  |  |  |
| 13CZ3WF31M | M | RL | | 0 | 144 | | 0 | | 262 | 287 | | 301 | | 207 | 197 | | 0 | 188 | | 136 | 120 | 150 | | 140 | 0 | | 0 |  |  |  |  |  |  |  |  |  |
| 13CZ3WF11F | F | RR | | 0 | 0 | | 0 | | 0 | 287 | | 293 | | 207 | 251 | | 0 | 0 | | 136 | 140 | 146 | | 150 | 0 | | 0 |  |  |  |  |  |  |  |  |  |
| 39_2013JUV10 | F | RR | | 0 | 0 | | 0 | | 0 | 287 | | 293 | | 207 | 251 | | 0 | 0 | | 136 | 136 | 150 | | 150 | 0 | | 0 |  |  |  |  |  |  |  |  |  |
| 39_2013JUV11 | F | RR | | 0 | 0 | | 0 | | 0 | 287 | | 293 | | 207 | 251 | | 0 | 0 | | 136 | 140 | 150 | | 150 | 0 | | 0 |  |  |  |  |  |  |  |  |  |
| 39_2013JUV13 | F | RR | | 0 | 0 | | 0 | | 0 | 287 | | 293 | | 207 | 207 | | 0 | 0 | | 136 | 140 | 150 | | 150 | 0 | | 0 |  |  |  |  |  |  |  |  |  |
| 39_2013JUV14 | F | RR | | 0 | 0 | | 0 | | 0 | 287 | | 287 | | 207 | 207 | | 0 | 0 | | 136 | 136 | 150 | | 146 | 0 | | 0 |  |  |  |  |  |  |  |  |  |
| 39_2013JUV15 | F | RR | | 0 | 0 | | 0 | | 0 | 287 | | 287 | | 207 | 251 | | 0 | 0 | | 136 | 136 | 150 | | 146 | 0 | | 0 |  |  |  |  |  |  |  |  |  |
| 39_2013JUV16 | F | RR | | 0 | 0 | | 0 | | 0 | 287 | | 293 | | 207 | 207 | | 0 | 0 | | 136 | 140 | 150 | | 150 | 0 | | 0 |  |  |  |  |  |  |  |  |  |
| 39_2013JUV17 | F | RR | | 0 | 0 | | 0 | | 0 | 287 | | 287 | | 207 | 207 | | 0 | 0 | | 136 | 136 | 150 | | 146 | 0 | | 0 |  |  |  |  |  |  |  |  |  |
| 39_2013JUV18 | F | RR | | 0 | 0 | | 0 | | 0 | 287 | | 287 | | 207 | 251 | | 0 | 0 | | 136 | 140 | 150 | | 150 | 0 | | 0 |  |  |  |  |  |  |  |  |  |
| 39_2013JUV19 | F | RR | | 0 | 0 | | 0 | | 0 | 287 | | 287 | | 207 | 251 | | 0 | 0 | | 136 | 140 | 150 | | 150 | 0 | | 0 |  |  |  |  |  |  |  |  |  |
| 39_2013JUV1B | F | RR | | 0 | 0 | | 0 | | 0 | 287 | | 293 | | 207 | 207 | | 0 | 0 | | 136 | 136 | 150 | | 150 | 0 | | 0 |  |  |  |  |  |  |  |  |  |
| 39_2013JUV1 | F | RR | | 0 | 0 | | 0 | | 0 | 287 | | 293 | | 207 | 251 | | 0 | 0 | | 136 | 136 | 150 | | 146 | 0 | | 0 |  |  |  |  |  |  |  |  |  |
| 39_2013JUV20 | F | RR | | 0 | 0 | | 0 | | 0 | 287 | | 293 | | 207 | 251 | | 0 | 0 | | 136 | 136 | 150 | | 150 | 0 | | 0 |  |  |  |  |  |  |  |  |  |
| 39_2013JUV2B | F | RR | | 0 | 0 | | 0 | | 0 | 287 | | 293 | | 207 | 207 | | 0 | 0 | | 136 | 140 | 150 | | 146 | 0 | | 0 |  |  |  |  |  |  |  |  |  |
| 39_2013JUV2 | F | RR | | 0 | 0 | | 0 | | 0 | 287 | | 293 | | 207 | 251 | | 0 | 0 | | 136 | 140 | 150 | | 150 | 0 | | 0 |  |  |  |  |  |  |  |  |  |
| 39_2013JUV3B | F | RR | | 0 | 0 | | 0 | | 0 | 287 | | 293 | | 207 | 251 | | 0 | 0 | | 136 | 140 | 150 | | 150 | 0 | | 0 |  |  |  |  |  |  |  |  |  |
| 39_2013JUV4B | F | RR | | 0 | 0 | | 0 | | 0 | 287 | | 287 | | 207 | 207 | | 0 | 0 | | 136 | 136 | 150 | | 146 | 0 | | 0 |  |  |  |  |  |  |  |  |  |
| 39_2013JUV5B | F | RR | | 0 | 0 | | 0 | | 0 | 287 | | 287 | | 207 | 207 | | 0 | 0 | | 136 | 136 | 150 | | 150 | 0 | | 0 |  |  |  |  |  |  |  |  |  |
| 39_2013JUV6B | F | RR | | 0 | 0 | | 0 | | 0 | 287 | | 287 | | 207 | 207 | | 0 | 0 | | 136 | 136 | 150 | | 150 | 0 | | 0 |  |  |  |  |  |  |  |  |  |
| 39_2013JUV7B | F | RR | | 0 | 0 | | 0 | | 0 | 287 | | 287 | | 207 | 207 | | 0 | 0 | | 136 | 140 | 150 | | 146 | 0 | | 0 |  |  |  |  |  |  |  |  |  |
| 39_2013JUV8B | F | RR | | 0 | 0 | | 0 | | 0 | 287 | | 287 | | 207 | 251 | | 0 | 0 | | 136 | 140 | 150 | | 150 | 0 | | 0 |  |  |  |  |  |  |  |  |  |
| 39_2013JUV9B | F | RR | | 0 | 0 | | 0 | | 0 | 287 | | 293 | | 207 | 207 | | 0 | 0 | | 136 | 136 | 150 | | 150 | 0 | | 0 |  |  |  |  |  |  |  |  |  |
| 13CZ3WF38M | M | RL | | 0 | 144 | | 0 | | 262 | 287 | | 301 | | 207 | 197 | | 0 | 188 | | 136 | 120 | 146 | | 140 | 0 | | 124 |  |  |  |  |  |  |  |  |  |
| 13CZ3WF11F | F | RR | | 0 | 0 | | 0 | | 0 | 287 | | 293 | | 207 | 251 | | 0 | 0 | | 136 | 140 | 146 | | 150 | 0 | | 0 |  |  |  |  |  |  |  |  |  |
| 41_2013JUV10 | M | RL | | 0 | 144 | | 0 | | 262 | 293 | | 301 | | 207 | 197 | | 0 | 188 | | 140 | 120 | 146 | | 140 | 0 | | 124 |  |  |  |  |  |  |  |  |  |
| 41_2013JUV11 | M | RL | | 0 | 144 | | 0 | | 262 | 287 | | 301 | | 251 | 197 | | 0 | 188 | | 136 | 120 | 150 | | 140 | 0 | | 124 |  |  |  |  |  |  |  |  |  |
| 41_2013JUV12 | M | RL | | 0 | 144 | | 0 | | 262 | 287 | | 301 | | 251 | 197 | | 0 | 188 | | 136 | 120 | 150 | | 140 | 0 | | 124 |  |  |  |  |  |  |  |  |  |
| 41_2013JUV13 | M | RL | | 0 | 144 | | 0 | | 262 | 293 | | 301 | | 251 | 197 | | 0 | 188 | | 140 | 120 | 150 | | 140 | 0 | | 124 |  |  |  |  |  |  |  |  |  |
| 41_2013JUV14 | M | RL | | 0 | 144 | | 0 | | 262 | 293 | | 301 | | 251 | 197 | | 0 | 188 | | 140 | 120 | 146 | | 140 | 0 | | 124 |  |  |  |  |  |  |  |  |  |
| 41_2013JUV15 | M | RL | | 0 | 144 | | 0 | | 262 | 287 | | 301 | | 207 | 197 | | 0 | 188 | | 136 | 120 | 150 | | 140 | 0 | | 124 |  |  |  |  |  |  |  |  |  |
| 41_2013JUV16 | M | RL | | 0 | 144 | | 0 | | 262 | 0 | | 0 | | 0 | 197 | | 0 | 0 | | 0 | 120 | 150 | | 140 | 0 | | 0 |  |  |  |  |  |  |  |  |  |
| 41_2013JUV17 | M | RL | | 0 | 144 | | 0 | | 262 | 287 | | 301 | | 207 | 197 | | 0 | 0 | | 136 | 120 | 150 | | 140 | 0 | | 0 |  |  |  |  |  |  |  |  |  |
| 41_2013JUV18 | M | RL | | 0 | 144 | | 0 | | 262 | 287 | | 301 | | 207 | 197 | | 0 | 188 | | 140 | 120 | 150 | | 140 | 0 | | 0 |  |  |  |  |  |  |  |  |  |
| 41_2013JUV19 | M | RL | | 0 | 144 | | 0 | | 262 | 287 | | 301 | | 207 | 197 | | 0 | 188 | | 140 | 120 | 146 | | 140 | 0 | | 0 |  |  |  |  |  |  |  |  |  |
| 41_2013JUV20 | M | RL | | 0 | 144 | | 0 | | 262 | 287 | | 301 | | 207 | 197 | | 0 | 188 | | 140 | 120 | 150 | | 140 | 0 | | 0 |  |  |  |  |  |  |  |  |  |
| 41_2013JUV9 | M | RL | | 0 | 144 | | 0 | | 262 | 293 | | 301 | | 251 | 197 | | 0 | 188 | | 140 | 120 | 150 | | 140 | 0 | | 124 |  |  |  |  |  |  |  |  |  |
| 41_2013JUV1 | M | RL | | 0 | 144 | | 0 | | 262 | 287 | | 301 | | 207 | 197 | | 0 | 188 | | 140 | 120 | 150 | | 140 | 0 | | 124 |  |  |  |  |  |  |  |  |  |
| 41_2013JUV2 | M | RL | | 0 | 144 | | 0 | | 262 | 293 | | 301 | | 251 | 197 | | 0 | 188 | | 136 | 120 | 150 | | 140 | 0 | | 124 |  |  |  |  |  |  |  |  |  |
| 41_2013JUV3 | M | RL | | 0 | 144 | | 0 | | 262 | 293 | | 301 | | 207 | 197 | | 0 | 188 | | 136 | 120 | 146 | | 140 | 0 | | 124 |  |  |  |  |  |  |  |  |  |
| 41_2013JUV4 | M | RL | | 0 | 144 | | 0 | | 262 | 287 | | 301 | | 207 | 197 | | 0 | 188 | | 140 | 120 | 146 | | 140 | 0 | | 124 |  |  |  |  |  |  |  |  |  |
| 41_2013JUV5 | M | RL | | 0 | 144 | | 0 | | 262 | 287 | | 301 | | 251 | 197 | | 0 | 188 | | 136 | 120 | 146 | | 140 | 0 | | 124 |  |  |  |  |  |  |  |  |  |
| 41_2013JUV6 | M | RL | | 0 | 144 | | 0 | | 262 | 293 | | 301 | | 251 | 197 | | 0 | 188 | | 140 | 120 | 150 | | 140 | 0 | | 124 |  |  |  |  |  |  |  |  |  |
| 41_2013JUV7 | M | RL | | 0 | 144 | | 0 | | 262 | 287 | | 301 | | 207 | 197 | | 0 | 188 | | 136 | 120 | 150 | | 140 | 0 | | 124 |  |  |  |  |  |  |  |  |  |
| 41_2013JUV8 | M | RL | | 0 | 144 | | 0 | | 262 | 293 | | 301 | | 251 | 197 | | 0 | 188 | | 136 | 120 | 150 | | 140 | 0 | | 124 |  |  |  |  |  |  |  |  |  |
| 13CZ3WF32M | M | RL | | 0 | 144 | | 0 | | 262 | 287 | | 301 | | 211 | 197 | | 0 | 188 | | 136 | 120 | 0 | | 140 | 0 | | 124 |  |  |  |  |  |  |  |  |  |
| 13CZ3WF11F | F | RR | | 0 | 0 | | 0 | | 0 | 287 | | 293 | | 207 | 251 | | 0 | 0 | | 136 | 140 | 146 | | 150 | 0 | | 0 |  |  |  |  |  |  |  |  |  |
| 42_2013JUV1 | M | RL | | 0 | 144 | | 0 | | 262 | 293 | | 301 | | 251 | 197 | | 0 | 188 | | 136 | 120 | 150 | | 140 | 0 | | 124 |  |  |  |  |  |  |  |  |  |
| 42_2013JUV1B | M | RL | | 0 | 144 | | 0 | | 262 | 293 | | 301 | | 207 | 197 | | 0 | 188 | | 136 | 120 | 150 | | 140 | 0 | | 124 |  |  |  |  |  |  |  |  |  |
| 42_2013JUV2 | M | RL | | 0 | 144 | | 0 | | 262 | 293 | | 301 | | 251 | 197 | | 0 | 188 | | 136 | 120 | 150 | | 140 | 0 | | 124 |  |  |  |  |  |  |  |  |  |
| 42_2013JUV2B | M | RL | | 0 | 144 | | 0 | | 262 | 293 | | 301 | | 251 | 197 | | 0 | 188 | | 136 | 120 | 146 | | 140 | 0 | | 124 |  |  |  |  |  |  |  |  |  |
| 42_2013JUV3 | M | RL | | 0 | 144 | | 0 | | 262 | 287 | | 301 | | 207 | 197 | | 0 | 188 | | 136 | 120 | 146 | | 140 | 0 | | 124 |  |  |  |  |  |  |  |  |  |
| 42_2013JUV3B | M | RL | | 0 | 144 | | 0 | | 262 | 287 | | 301 | | 207 | 197 | | 0 | 188 | | 140 | 120 | 146 | | 140 | 0 | | 124 |  |  |  |  |  |  |  |  |  |
| 42_2013JUV4 | M | RL | | 0 | 144 | | 0 | | 262 | 287 | | 301 | | 207 | 197 | | 0 | 188 | | 140 | 120 | 150 | | 140 | 0 | | 124 |  |  |  |  |  |  |  |  |  |
| 42_2013JUV4B | M | RL | | 0 | 144 | | 0 | | 262 | 293 | | 301 | | 207 | 197 | | 0 | 188 | | 136 | 120 | 146 | | 140 | 0 | | 124 |  |  |  |  |  |  |  |  |  |
| 42_2013JUV5 | M | RL | | 0 | 144 | | 0 | | 262 | 293 | | 301 | | 207 | 197 | | 0 | 188 | | 136 | 120 | 146 | | 140 | 0 | | 124 |  |  |  |  |  |  |  |  |  |
| 42_2013JUV5B | M | RL | | 0 | 144 | | 0 | | 262 | 287 | | 301 | | 251 | 197 | | 0 | 188 | | 136 | 120 | 146 | | 140 | 0 | | 124 |  |  |  |  |  |  |  |  |  |
| 42_2013JUV6 | M | RL | | 0 | 144 | | 0 | | 262 | 293 | | 301 | | 207 | 197 | | 0 | 188 | | 140 | 120 | 150 | | 140 | 0 | | 124 |  |  |  |  |  |  |  |  |  |
| 42_2013JUV6B | M | RL | | 0 | 144 | | 0 | | 262 | 293 | | 301 | | 251 | 197 | | 0 | 188 | | 136 | 120 | 150 | | 140 | 0 | | 124 |  |  |  |  |  |  |  |  |  |
| 42_2013JUV7B | M | RL | | 0 | 144 | | 0 | | 262 | 293 | | 301 | | 251 | 197 | | 0 | 188 | | 140 | 120 | 150 | | 140 | 0 | | 124 |  |  |  |  |  |  |  |  |  |
| 42_2013JUV8B | M | RL | | 0 | 144 | | 0 | | 262 | 293 | | 301 | | 251 | 197 | | 0 | 188 | | 140 | 120 | 150 | | 140 | 0 | | 124 |  |  |  |  |  |  |  |  |  |
| 42_2013JUV9B | M | RL | | 0 | 0 | | 0 | | 0 | 0 | | 0 | | 0 | 0 | | 0 | 0 | | 0 | 0 | 150 | | 140 | 0 | | 0 |  |  |  |  |  |  |  |  |  |
| 13CZ3WF31M | M | RL | | 0 | 144 | | 0 | | 262 | 287 | | 301 | | 207 | 197 | | 0 | 188 | | 136 | 120 | 150 | | 140 | 0 | | 0 |  |  |  |  |  |  |  |  |  |
| 13CZ1WF4F | F | RR | | 0 | 0 | | 0 | | 0 | 287 | | 287 | | 207 | 224 | | 0 | 0 | | 136 | 136 | 146 | | 150 | 0 | | 0 |  |  |  |  |  |  |  |  |  |
| 49_2013JUV1 | M | RL | | 0 | 144 | | 0 | | 262 | 287 | | 301 | | 0 | 0 | | 0 | 188 | | 136 | 120 | 146 | | 140 | 0 | | 124 |  |  |  |  |  |  |  |  |  |
| 49_2013JUV2 | F | RR | | 0 | 0 | | 0 | | 0 | 287 | | 287 | | 224 | 207 | | 0 | 0 | | 136 | 136 | 146 | | 150 | 0 | | 0 |  |  |  |  |  |  |  |  |  |
| 13CZ3WF36M | M | RL | | 0 | 144 | | 0 | | 262 | 287 | | 301 | | 207 | 197 | | 0 | 188 | | 136 | 120 | 150 | | 140 | 0 | | 124 |  |  |  |  |  |  |  |  |  |
| 13CZ4WF1F | F | RR | | 0 | 0 | | 0 | | 0 | 287 | | 287 | | 203 | 249 | | 0 | 0 | | 140 | 136 | 150 | | 146 | 0 | | 0 |  |  |  |  |  |  |  |  |  |
| 53_2013JUV1 | M | RL | | 0 | 144 | | 0 | | 262 | 287 | | 301 | | 249 | 197 | | 0 | 0 | | 136 | 120 | 146 | | 140 | 0 | | 124 |  |  |  |  |  |  |  |  |  |
| 53_2013JUV2 | M | RL | | 0 | 144 | | 0 | | 262 | 287 | | 301 | | 203 | 197 | | 0 | 188 | | 136 | 120 | 150 | | 140 | 0 | | 124 |  |  |  |  |  |  |  |  |  |
| 13CZ3WF44M | M | RL | | 0 | 144 | | 0 | | 262 | 287 | | 301 | | 203 | 197 | | 0 | 188 | | 136 | 120 | 146 | | 140 | 0 | | 124 |  |  |  |  |  |  |  |  |  |
| 13CZ4WF1F | F | RR | | 0 | 0 | | 0 | | 0 | 287 | | 287 | | 203 | 249 | | 0 | 0 | | 140 | 136 | 150 | | 146 | 0 | | 0 |  |  |  |  |  |  |  |  |  |
| 54_2013JUV1 | M | RL | | 0 | 144 | | 0 | | 262 | 287 | | 301 | | 0 | 0 | | 0 | 188 | | 136 | 120 | 150 | | 140 | 0 | | 124 |  |  |  |  |  |  |  |  |  |
| 54_2013JUV2 | M | RL | | 0 | 0 | | 0 | | 0 | 0 | | 0 | | 0 | 0 | | 0 | 0 | | 0 | 0 | 0 | | 0 | 0 | | 0 |  |  |  |  |  |  |  |  |  |
| 13CZ3WF39M | M | RL | | 0 | 144 | | 0 | | 262 | 287 | | 301 | | 207 | 197 | | 0 | 188 | | 134 | 120 | 150 | | 140 | 0 | | 124 |  |  |  |  |  |  |  |  |  |
| 13CZ4WF1F | F | RR | | 0 | 0 | | 0 | | 0 | 287 | | 287 | | 203 | 249 | | 0 | 0 | | 140 | 136 | 150 | | 146 | 0 | | 0 |  |  |  |  |  |  |  |  |  |
| 57_2013JUV1 | M | RL | | 0 | 144 | | 0 | | 262 | 287 | | 301 | | 0 | 0 | | 0 | 188 | | 136 | 120 | 150 | | 140 | 0 | | 124 |  |  |  |  |  |  |  |  |  |
| 57_2013JUV2 | M | RL | | 0 | 144 | | 0 | | 262 | 287 | | 301 | | 0 | 0 | | 0 | 188 | | 136 | 120 | 150 | | 140 | 0 | | 124 |  |  |  |  |  |  |  |  |  |
| 57_2013JUV3 | M | RL | | 0 | 144 | | 0 | | 262 | 287 | | 301 | | 0 | 0 | | 0 | 188 | | 136 | 120 | 150 | | 140 | 0 | | 124 |  |  |  |  |  |  |  |  |  |
| 13CZ3WF42M | M | RL | | 0 | 144 | | 0 | | 262 | 287 | | 301 | | 211 | 197 | | 0 | 188 | | 136 | 120 | 150 | | 140 | 0 | | 124 |  |  |  |  |  |  |  |  |  |
| 13CZ4WF1F | F | RR | | 0 | 0 | | 0 | | 0 | 287 | | 287 | | 203 | 249 | | 0 | 0 | | 140 | 136 | 150 | | 146 | 0 | | 0 |  |  |  |  |  |  |  |  |  |
| 59_2013JUV1 | F | RR | | 0 | 0 | | 0 | | 0 | 287 | | 287 | | 203 | 211 | | 0 | 0 | | 136 | 136 | 150 | | 150 | 0 | | 0 |  |  |  |  |  |  |  |  |  |
| 59_2013JUV2 | F | RR | | 0 | 0 | | 0 | | 0 | 287 | | 287 | | 203 | 211 | | 0 | 0 | | 136 | 136 | 150 | | 150 | 0 | | 0 |  |  |  |  |  |  |  |  |  |
| 59_2013JUV3 | F | RR | | 0 | 0 | | 0 | | 0 | 287 | | 287 | | 0 | 211 | | 0 | 0 | | 140 | 136 | 150 | | 150 | 0 | | 0 |  |  |  |  |  |  |  |  |  |
| 13CZ3WF43M | M | RL | | 0 | 144 | | 0 | | 262 | 287 | | 301 | | 207 | 197 | | 0 | 188 | | 136 | 120 | 150 | | 140 | 0 | | 124 |  |  |  |  |  |  |  |  |  |
| 13CZ4WF1F | F | RR | | 0 | 0 | | 0 | | 0 | 287 | | 287 | | 203 | 249 | | 0 | 0 | | 140 | 136 | 150 | | 146 | 0 | | 0 |  |  |  |  |  |  |  |  |  |
| 60_2013JUV10 | M | RL | | 0 | 144 | | 0 | | 262 | 287 | | 301 | | 0 | 0 | | 0 | 188 | | 136 | 120 | 150 | | 140 | 0 | | 124 |  |  |  |  |  |  |  |  |  |
| 60_2013JUV11 | M | RL | | 0 | 144 | | 0 | | 262 | 287 | | 301 | | 249 | 197 | | 0 | 188 | | 140 | 120 | 150 | | 140 | 0 | | 124 |  |  |  |  |  |  |  |  |  |
| 60_2013JUV12 | M | RL | | 0 | 144 | | 0 | | 262 | 287 | | 301 | | 249 | 197 | | 0 | 188 | | 136 | 120 | 146 | | 140 | 0 | | 124 |  |  |  |  |  |  |  |  |  |
| 60_2013JUV13 | M | RL | | 0 | 144 | | 0 | | 262 | 287 | | 301 | | 203 | 197 | | 0 | 188 | | 140 | 120 | 146 | | 140 | 0 | | 124 |  |  |  |  |  |  |  |  |  |
| 60_2013JUV14 | M | RL | | 0 | 144 | | 0 | | 262 | 287 | | 301 | | 203 | 197 | | 0 | 188 | | 136 | 120 | 146 | | 140 | 0 | | 124 |  |  |  |  |  |  |  |  |  |
| 60_2013JUV16 | M | RL | | 0 | 144 | | 0 | | 262 | 287 | | 301 | | 203 | 197 | | 0 | 188 | | 140 | 120 | 150 | | 140 | 0 | | 124 |  |  |  |  |  |  |  |  |  |
| 60_2013JUV17 | M | RL | | 0 | 144 | | 0 | | 262 | 287 | | 301 | | 249 | 197 | | 0 | 188 | | 140 | 120 | 150 | | 140 | 0 | | 124 |  |  |  |  |  |  |  |  |  |
| 60_2013JUV18 | M | RL | | 0 | 144 | | 0 | | 262 | 287 | | 301 | | 203 | 197 | | 0 | 188 | | 136 | 120 | 146 | | 140 | 0 | | 0 |  |  |  |  |  |  |  |  |  |
| 60_2013JUV19 | M | RL | | 0 | 144 | | 0 | | 262 | 287 | | 301 | | 249 | 197 | | 0 | 188 | | 136 | 120 | 150 | | 140 | 0 | | 0 |  |  |  |  |  |  |  |  |  |
| 60_2013JUV2 | M | RL | | 0 | 144 | | 0 | | 262 | 287 | | 301 | | 249 | 197 | | 0 | 188 | | 140 | 120 | 150 | | 140 | 0 | | 124 |  |  |  |  |  |  |  |  |  |
| 60_2013JUV20 | M | RL | | 0 | 144 | | 0 | | 262 | 287 | | 301 | | 203 | 197 | | 0 | 188 | | 140 | 120 | 150 | | 140 | 0 | | 124 |  |  |  |  |  |  |  |  |  |
| 60_2013JUV3 | M | RL | | 0 | 144 | | 0 | | 262 | 287 | | 301 | | 203 | 197 | | 0 | 188 | | 140 | 120 | 150 | | 140 | 0 | | 124 |  |  |  |  |  |  |  |  |  |
| 60_2013JUV4 | M | RL | | 0 | 144 | | 0 | | 262 | 287 | | 301 | | 0 | 0 | | 0 | 188 | | 136 | 120 | 150 | | 140 | 0 | | 124 |  |  |  |  |  |  |  |  |  |
| 60_2013JUV5 | M | RL | | 0 | 144 | | 0 | | 262 | 287 | | 301 | | 249 | 197 | | 0 | 0 | | 136 | 120 | 146 | | 140 | 0 | | 124 |  |  |  |  |  |  |  |  |  |
| 60_2013JUV6 | M | RL | | 0 | 144 | | 0 | | 262 | 287 | | 301 | | 203 | 197 | | 0 | 0 | | 0 | 120 | 146 | | 140 | 0 | | 124 |  |  |  |  |  |  |  |  |  |
| 60_2013JUV8 | M | RL | | 0 | 144 | | 0 | | 262 | 287 | | 301 | | 203 | 197 | | 0 | 188 | | 136 | 120 | 150 | | 140 | 0 | | 124 |  |  |  |  |  |  |  |  |  |
| 60_2013JUV9 | M | RL | | 0 | 144 | | 0 | | 262 | 287 | | 301 | | 0 | 0 | | 0 | 188 | | 136 | 120 | 146 | | 140 | 0 | | 124 |  |  |  |  |  |  |  |  |  |
| 60_2013JUV15 | M | RL | | 0 | 144 | | 0 | | 262 | 287 | | 301 | | 203 | 197 | | 0 | 188 | | 136 | 120 | 146 | | 140 | 0 | | 124 |  |  |  |  |  |  |  |  |  |
| 60_2013JUV1 | M | RL | | 0 | 144 | | 0 | | 262 | 287 | | 301 | | 249 | 197 | | 0 | 188 | | 140 | 120 | 146 | | 140 | 0 | | 124 |  |  |  |  |  |  |  |  |  |
| 13CZ3WF44M | M | RL | | 0 | 144 | | 0 | | 262 | 287 | | 301 | | 203 | 197 | | 0 | 188 | | 136 | 120 | 146 | | 140 | 0 | | 124 |  |  |  |  |  |  |  |  |  |
| 13CZ3WF3F | F | RR | | 0 | 0 | | 0 | | 0 | 287 | | 287 | | 207 | 211 | | 0 | 0 | | 136 | 136 | 146 | | 146 | 0 | | 0 |  |  |  |  |  |  |  |  |  |
| 63_2013JUV1 | M | RL | | 0 | 144 | | 0 | | 262 | 287 | | 301 | | 207 | 197 | | 0 | 188 | | 136 | 120 | 146 | | 140 | 0 | | 0 |  |  |  |  |  |  |  |  |  |
| 63_2013JUV2 | M | RL | | 0 | 144 | | 0 | | 262 | 287 | | 301 | | 207 | 197 | | 0 | 188 | | 136 | 120 | 146 | | 140 | 0 | | 0 |  |  |  |  |  |  |  |  |  |
| 63_2013JUV3 | M | RL | | 0 | 144 | | 0 | | 262 | 287 | | 301 | | 207 | 197 | | 0 | 188 | | 136 | 120 | 146 | | 140 | 0 | | 0 |  |  |  |  |  |  |  |  |  |
| 13CZ3WF30M | M | RL | | 0 | 144 | | 0 | | 262 | 287 | | 301 | | 207 | 197 | | 0 | 188 | | 136 | 120 | 146 | | 140 | 0 | | 124 |  |  |  |  |  |  |  |  |  |
| 13CZ3WF3F | F | RR | | 0 | 0 | | 0 | | 0 | 287 | | 287 | | 207 | 211 | | 0 | 0 | | 136 | 136 | 146 | | 146 | 0 | | 0 |  |  |  |  |  |  |  |  |  |
| 65_2013JUV1 | M | RL | | 0 | 144 | | 0 | | 262 | 287 | | 301 | | 207 | 197 | | 0 | 188 | | 136 | 120 | 146 | | 140 | 0 | | 0 |  |  |  |  |  |  |  |  |  |
| 13CZ3WF39M | M | RL | | 0 | 144 | | 0 | | 262 | 287 | | 301 | | 207 | 197 | | 0 | 188 | | 134 | 120 | 150 | | 140 | 0 | | 124 |  |  |  |  |  |  |  |  |  |
| 13CZ3WF3F | F | RR | | 0 | 0 | | 0 | | 0 | 287 | | 287 | | 207 | 211 | | 0 | 0 | | 136 | 136 | 146 | | 146 | 0 | | 0 |  |  |  |  |  |  |  |  |  |
| 66_2013JUV1 | M | RL | | 0 | 144 | | 0 | | 262 | 287 | | 301 | | 207 | 197 | | 0 | 188 | | 136 | 120 | 146 | | 140 | 0 | | 124 |  |  |  |  |  |  |  |  |  |
| 66_2013JUV2 | M | RL | | 0 | 144 | | 0 | | 262 | 287 | | 301 | | 211 | 197 | | 0 | 188 | | 136 | 120 | 146 | | 140 | 0 | | 0 |  |  |  |  |  |  |  |  |  |
| 66_2013JUV3 | M | RL | | 0 | 144 | | 0 | | 262 | 287 | | 301 | | 211 | 197 | | 0 | 188 | | 136 | 120 | 146 | | 140 | 0 | | 124 |  |  |  |  |  |  |  |  |  |
| 66_2013JUV4 | M | RL | | 0 | 144 | | 0 | | 262 | 287 | | 301 | | 211 | 197 | | 0 | 188 | | 136 | 120 | 146 | | 140 | 0 | | 124 |  |  |  |  |  |  |  |  |  |
| 66_2013JUV5 | M | RL | | 0 | 144 | | 0 | | 262 | 287 | | 301 | | 211 | 197 | | 0 | 188 | | 136 | 120 | 146 | | 140 | 0 | | 124 |  |  |  |  |  |  |  |  |  |
| 66_2013JUV6 | M | RL | | 0 | 144 | | 0 | | 262 | 287 | | 301 | | 211 | 197 | | 0 | 188 | | 136 | 120 | 146 | | 140 | 0 | | 124 |  |  |  |  |  |  |  |  |  |
| 66_2013JUV7 | M | RL | | 0 | 144 | | 0 | | 262 | 287 | | 301 | | 207 | 197 | | 0 | 188 | | 136 | 120 | 146 | | 140 | 0 | | 0 |  |  |  |  |  |  |  |  |  |
| 66_2013JUV10 | M | RL | | 0 | 144 | | 0 | | 262 | 287 | | 301 | | 211 | 197 | | 0 | 188 | | 136 | 120 | 146 | | 140 | 0 | | 124 |  |  |  |  |  |  |  |  |  |
| 66_2013JUV11 | M | RL | | 0 | 144 | | 0 | | 262 | 287 | | 301 | | 207 | 197 | | 0 | 188 | | 136 | 120 | 146 | | 140 | 0 | | 0 |  |  |  |  |  |  |  |  |  |
| 66_2013JUV12 | M | RL | | 0 | 144 | | 0 | | 262 | 287 | | 301 | | 211 | 197 | | 0 | 188 | | 136 | 120 | 146 | | 140 | 0 | | 0 |  |  |  |  |  |  |  |  |  |
| 66_2013JUV13 | M | RL | | 0 | 144 | | 0 | | 262 | 287 | | 301 | | 207 | 197 | | 0 | 188 | | 136 | 120 | 146 | | 140 | 0 | | 0 |  |  |  |  |  |  |  |  |  |
| 66_2013JUV14 | M | RL | | 0 | 144 | | 0 | | 262 | 287 | | 301 | | 211 | 197 | | 0 | 188 | | 136 | 120 | 146 | | 140 | 0 | | 124 |  |  |  |  |  |  |  |  |  |
| 66_2013JUV15 | M | RL | | 0 | 144 | | 0 | | 262 | 287 | | 301 | | 211 | 197 | | 0 | 188 | | 136 | 120 | 146 | | 140 | 0 | | 124 |  |  |  |  |  |  |  |  |  |
| 66_2013JUV16 | M | RL | | 0 | 144 | | 0 | | 262 | 287 | | 301 | | 207 | 197 | | 0 | 188 | | 136 | 120 | 146 | | 140 | 0 | | 0 |  |  |  |  |  |  |  |  |  |
| 66_2013JUV17 | M | RL | | 0 | 144 | | 0 | | 262 | 287 | | 301 | | 211 | 197 | | 0 | 188 | | 136 | 120 | 146 | | 140 | 0 | | 124 |  |  |  |  |  |  |  |  |  |
| 66_2013JUV18 | M | RL | | 0 | 144 | | 0 | | 262 | 287 | | 301 | | 211 | 197 | | 0 | 188 | | 136 | 120 | 146 | | 140 | 0 | | 124 |  |  |  |  |  |  |  |  |  |
| 66_2013JUV19 | M | RL | | 0 | 144 | | 0 | | 262 | 287 | | 301 | | 211 | 197 | | 0 | 188 | | 136 | 120 | 146 | | 140 | 0 | | 124 |  |  |  |  |  |  |  |  |  |
| 66_2013JUV20 | M | RL | | 0 | 144 | | 0 | | 262 | 287 | | 301 | | 207 | 197 | | 0 | 188 | | 136 | 120 | 146 | | 140 | 0 | | 0 |  |  |  |  |  |  |  |  |  |
| 66_2013JUV21 | M | RL | | 0 | 144 | | 0 | | 262 | 287 | | 301 | | 211 | 197 | | 0 | 188 | | 136 | 120 | 146 | | 140 | 0 | | 0 |  |  |  |  |  |  |  |  |  |
| 66_2013JUV22 | M | RL | | 0 | 144 | | 0 | | 262 | 287 | | 301 | | 207 | 197 | | 0 | 188 | | 136 | 120 | 146 | | 140 | 0 | | 0 |  |  |  |  |  |  |  |  |  |
| 66_2013JUV8 | M | RL | | 0 | 144 | | 0 | | 262 | 287 | | 301 | | 211 | 197 | | 0 | 188 | | 136 | 120 | 146 | | 140 | 0 | | 124 |  |  |  |  |  |  |  |  |  |
| 66_2013JUV9 | M | RL | | 0 | 144 | | 0 | | 262 | 287 | | 301 | | 0 | 0 | | 0 | 188 | | 136 | 120 | 146 | | 140 | 0 | | 124 |  |  |  |  |  |  |  |  |  |
| Multiplex 2 |  |  | | Microsatellite loci | | | | | | | | | | | | | | | | | | | | |  | |  |  |  |  |  |  |  |  |  |  |
| **ID** | **Sex** | **Gen** | | **Re1Caga10** | | | **Re2Caga3** | | | **RICA1b6** | | | | **Res22** | | | **Rrid059A** | | | **Rrid082A** | | **Rrid169A** | | |  | |  |  |  |  |  |  |  |  |  |  |
| 13CZ3WF28M | M | RL | | 127 | 0 | | 173 | | 0 | 103 | | 83 | | 113 | 0 | | 137 | 0 | | 163 | 0 | 192 | | 0 |  | |  |  |  |  |  |  |  |  |  |  |
| 13CZ1WF3F | F | RR | | 115 | 117 | | 223 | | 223 | 96 | | 88 | | 113 | 113 | | 131 | 135 | | 178 | 178 | 187 | | 197 |  | |  |  |  |  |  |  |  |  |  |  |
| 25_2013JUV1 | M | RL | | 115 | 0 | | 223 | | 0 | 96 | | 83 | | 113 | 0 | | 131 | 0 | | 178 | 0 | 187 | | 0 |  | |  |  |  |  |  |  |  |  |  |  |
| 25_2013JUV10 | M | RL | | 115 | 0 | | 223 | | 0 | 96 | | 83 | | 113 | 0 | | 135 | 0 | | 178 | 0 | 197 | | 0 |  | |  |  |  |  |  |  |  |  |  |  |
| 25_2013JUV11 | M | RL | | 115 | 0 | | 223 | | 0 | 96 | | 83 | | 113 | 0 | | 135 | 0 | | 178 | 0 | 197 | | 0 |  | |  |  |  |  |  |  |  |  |  |  |
| 25_2013JUV12 | M | RL | | 117 | 0 | | 223 | | 0 | 96 | | 83 | | 113 | 0 | | 0 | 0 | | 178 | 0 | 187 | | 0 |  | |  |  |  |  |  |  |  |  |  |  |
| 25_2013JUV13 | M | RL | | 0 | 0 | | 0 | | 0 | 0 | | 0 | | 0 | 0 | | 0 | 0 | | 0 | 0 | 0 | | 0 |  | |  |  |  |  |  |  |  |  |  |  |
| 25_2013JUV14 | M | RL | | 115 | 0 | | 223 | | 0 | 96 | | 83 | | 113 | 0 | | 135 | 0 | | 178 | 0 | 197 | | 0 |  | |  |  |  |  |  |  |  |  |  |  |
| 25_2013JUV15 | M | RL | | 0 | 0 | | 0 | | 0 | 0 | | 0 | | 0 | 0 | | 0 | 0 | | 0 | 0 | 0 | | 0 |  | |  |  |  |  |  |  |  |  |  |  |
| 25_2013JUV16 | M | RL | | 0 | 0 | | 0 | | 0 | 0 | | 0 | | 0 | 0 | | 0 | 0 | | 0 | 0 | 0 | | 0 |  | |  |  |  |  |  |  |  |  |  |  |
| 25_2013JUV17 | M | RL | | 0 | 0 | | 0 | | 0 | 0 | | 0 | | 0 | 0 | | 0 | 0 | | 0 | 0 | 0 | | 0 |  | |  |  |  |  |  |  |  |  |  |  |
| 25_2013JUV18 | M | RL | | 0 | 0 | | 0 | | 0 | 0 | | 0 | | 0 | 0 | | 0 | 0 | | 0 | 0 | 0 | | 0 |  | |  |  |  |  |  |  |  |  |  |  |
| 25_2013JUV19 | M | RL | | 0 | 0 | | 0 | | 0 | 0 | | 0 | | 0 | 0 | | 0 | 0 | | 0 | 0 | 0 | | 0 |  | |  |  |  |  |  |  |  |  |  |  |
| 25_2013JUV2 | M | RL | | 117 | 0 | | 223 | | 0 | 96 | | 83 | | 113 | 0 | | 135 | 0 | | 178 | 0 | 197 | | 0 |  | |  |  |  |  |  |  |  |  |  |  |
| 25_2013JUV20 | M | RL | | 0 | 0 | | 0 | | 0 | 0 | | 0 | | 0 | 0 | | 0 | 0 | | 0 | 0 | 0 | | 0 |  | |  |  |  |  |  |  |  |  |  |  |
| 25_2013JUV3 | M | RL | | 117 | 0 | | 223 | | 0 | 96 | | 83 | | 113 | 0 | | 135 | 0 | | 178 | 0 | 197 | | 0 |  | |  |  |  |  |  |  |  |  |  |  |
| 25_2013JUV4 | M | RL | | 117 | 0 | | 223 | | 0 | 96 | | 83 | | 113 | 0 | | 135 | 0 | | 178 | 0 | 197 | | 0 |  | |  |  |  |  |  |  |  |  |  |  |
| 25_2013JUV5 | M | RL | | 115 | 0 | | 223 | | 0 | 96 | | 83 | | 113 | 0 | | 135 | 0 | | 178 | 0 | 197 | | 0 |  | |  |  |  |  |  |  |  |  |  |  |
| 25_2013JUV6 | M | RL | | 115 | 0 | | 223 | | 0 | 96 | | 83 | | 113 | 0 | | 135 | 0 | | 178 | 0 | 187 | | 0 |  | |  |  |  |  |  |  |  |  |  |  |
| 25_2013JUV7 | M | RL | | 117 | 0 | | 223 | | 0 | 96 | | 83 | | 113 | 0 | | 135 | 0 | | 178 | 0 | 197 | | 0 |  | |  |  |  |  |  |  |  |  |  |  |
| 25_2013JUV8 | M | RL | | 115 | 0 | | 223 | | 0 | 96 | | 83 | | 113 | 0 | | 135 | 0 | | 178 | 0 | 197 | | 0 |  | |  |  |  |  |  |  |  |  |  |  |
| 25_2013JUV9 | M | RL | | 117 | 0 | | 223 | | 0 | 96 | | 83 | | 113 | 0 | | 131 | 0 | | 178 | 0 | 187 | | 0 |  | |  |  |  |  |  |  |  |  |  |  |
| 13CZ5WF1M | M | RL | | 106 | 0 | | 173 | | 0 | 96 | | 83 | | 106 | 0 | | 135 | 0 | | 163 | 0 | 195 | | 0 |  | |  |  |  |  |  |  |  |  |  |  |
| 13CZ1WF3F | F | RR | | 115 | 117 | | 223 | | 223 | 96 | | 88 | | 113 | 113 | | 131 | 135 | | 178 | 178 | 187 | | 197 |  | |  |  |  |  |  |  |  |  |  |  |
| 26_2013JUV1 | M | RL | | 115 | 0 | | 223 | | 0 | 0 | | 0 | | 113 | 0 | | 135 | 0 | | 178 | 0 | 187 | | 0 |  | |  |  |  |  |  |  |  |  |  |  |
| 26_2013JUV2 | M | RL | | 117 | 0 | | 223 | | 0 | 0 | | 0 | | 113 | 0 | | 135 | 0 | | 178 | 0 | 197 | | 0 |  | |  |  |  |  |  |  |  |  |  |  |
| 26_2013JUV3 | M | RL | | 115 | 0 | | 223 | | 0 | 96 | | 83 | | 113 | 0 | | 135 | 0 | | 178 | 0 | 187 | | 0 |  | |  |  |  |  |  |  |  |  |  |  |
| 26_2013JUV10 | M | RL | | 115 | 0 | | 223 | | 0 | 96 | | 83 | | 113 | 0 | | 135 | 0 | | 178 | 0 | 197 | | 0 |  | |  |  |  |  |  |  |  |  |  |  |
| 26_2013JUV11 | M | RL | | 115 | 0 | | 223 | | 0 | 96 | | 83 | | 113 | 0 | | 0 | 0 | | 178 | 0 | 197 | | 0 |  | |  |  |  |  |  |  |  |  |  |  |
| 26_2013JUV12 | M | RL | | 117 | 0 | | 223 | | 0 | 96 | | 83 | | 113 | 0 | | 0 | 0 | | 178 | 0 | 197 | | 0 |  | |  |  |  |  |  |  |  |  |  |  |
| 26_2013JUV13 | M | RL | | 115 | 0 | | 223 | | 0 | 96 | | 83 | | 113 | 0 | | 0 | 0 | | 178 | 0 | 187 | | 0 |  | |  |  |  |  |  |  |  |  |  |  |
| 26_2013JUV14 | M | RL | | 115 | 0 | | 223 | | 0 | 96 | | 83 | | 113 | 0 | | 0 | 0 | | 178 | 0 | 187 | | 0 |  | |  |  |  |  |  |  |  |  |  |  |
| 26_2013JUV15 | M | RL | | 117 | 0 | | 223 | | 0 | 96 | | 83 | | 113 | 0 | | 0 | 0 | | 178 | 0 | 187 | | 0 |  | |  |  |  |  |  |  |  |  |  |  |
| 26_2013JUV16 | M | RL | | 115 | 0 | | 223 | | 0 | 96 | | 83 | | 113 | 0 | | 0 | 0 | | 178 | 0 | 197 | | 0 |  | |  |  |  |  |  |  |  |  |  |  |
| 26_2013JUV4 | M | RL | | 117 | 0 | | 223 | | 0 | 96 | | 83 | | 113 | 0 | | 135 | 0 | | 178 | 0 | 187 | | 0 |  | |  |  |  |  |  |  |  |  |  |  |
| 26_2013JUV5 | M | RL | | 117 | 0 | | 223 | | 0 | 96 | | 83 | | 113 | 0 | | 135 | 0 | | 178 | 0 | 197 | | 0 |  | |  |  |  |  |  |  |  |  |  |  |
| 26_2013JUV6 | M | RL | | 115 | 0 | | 223 | | 0 | 96 | | 83 | | 113 | 0 | | 131 | 0 | | 178 | 0 | 187 | | 0 |  | |  |  |  |  |  |  |  |  |  |  |
| 26_2013JUV7 | M | RL | | 117 | 0 | | 223 | | 0 | 96 | | 83 | | 113 | 0 | | 135 | 0 | | 178 | 0 | 187 | | 0 |  | |  |  |  |  |  |  |  |  |  |  |
| 26_2013JUV8 | M | RL | | 117 | 0 | | 223 | | 0 | 96 | | 83 | | 113 | 0 | | 135 | 0 | | 178 | 0 | 197 | | 0 |  | |  |  |  |  |  |  |  |  |  |  |
| 26_2013JUV9 | M | RL | | 115 | 0 | | 223 | | 0 | 96 | | 83 | | 113 | 0 | | 131 | 0 | | 178 | 0 | 197 | | 0 |  | |  |  |  |  |  |  |  |  |  |  |
| 13CZ3WF40M | M | RL | | 127 | 0 | | 239 | | 0 | 98 | | 83 | | 85 | 0 | | 135 | 0 | | 178 | 0 | 189 | | 0 |  | |  |  |  |  |  |  |  |  |  |  |
| 13CZ1WF3F | F | RR | | 115 | 117 | | 223 | | 223 | 96 | | 88 | | 113 | 113 | | 131 | 135 | | 178 | 178 | 187 | | 197 |  | |  |  |  |  |  |  |  |  |  |  |
| 27_2013JUV10 | M | RL | | 117 | 0 | | 223 | | 0 | 96 | | 83 | | 113 | 0 | | 131 | 0 | | 178 | 0 | 187 | | 0 |  | |  |  |  |  |  |  |  |  |  |  |
| 27_2013JUV11 | F | RR | | 115 | 127 | | 223 | | 239 | 98 | | 96 | | 85 | 113 | | 131 | 135 | | 178 | 178 | 187 | | 189 |  | |  |  |  |  |  |  |  |  |  |  |
| 27_2013JUV12 | F | RR | | 117 | 127 | | 223 | | 239 | 98 | | 96 | | 85 | 113 | | 131 | 135 | | 178 | 178 | 197 | | 189 |  | |  |  |  |  |  |  |  |  |  |  |
| 27_2013JUV13 | F | RR | | 117 | 127 | | 223 | | 239 | 98 | | 96 | | 85 | 113 | | 0 | 0 | | 178 | 178 | 187 | | 189 |  | |  |  |  |  |  |  |  |  |  |  |
| 27_2013JUV14 | F | RR | | 115 | 127 | | 223 | | 239 | 98 | | 96 | | 85 | 113 | | 0 | 0 | | 178 | 178 | 187 | | 189 |  | |  |  |  |  |  |  |  |  |  |  |
| 27_2013JUV15 | F | RR | | 115 | 127 | | 0 | | 0 | 98 | | 96 | | 85 | 113 | | 0 | 0 | | 178 | 178 | 197 | | 189 |  | |  |  |  |  |  |  |  |  |  |  |
| 27_2013JUV16 | F | RR | | 115 | 127 | | 223 | | 239 | 98 | | 96 | | 85 | 113 | | 0 | 0 | | 178 | 178 | 197 | | 189 |  | |  |  |  |  |  |  |  |  |  |  |
| 27_2013JUV17 | M | RL | | 117 | 0 | | 223 | | 0 | 96 | | 83 | | 113 | 0 | | 0 | 0 | | 178 | 0 | 197 | | 0 |  | |  |  |  |  |  |  |  |  |  |  |
| 27_2013JUV18 | F | RR | | 115 | 127 | | 223 | | 239 | 98 | | 96 | | 85 | 113 | | 0 | 0 | | 178 | 178 | 187 | | 189 |  | |  |  |  |  |  |  |  |  |  |  |
| 27_2013JUV1 | M | RL | | 115 | 0 | | 223 | | 0 | 96 | | 83 | | 113 | 0 | | 135 | 0 | | 178 | 0 | 197 | | 0 |  | |  |  |  |  |  |  |  |  |  |  |
| 27_2013JUV21 | F | RR | | 115 | 127 | | 0 | | 0 | 98 | | 96 | | 85 | 113 | | 0 | 0 | | 178 | 178 | 187 | | 189 |  | |  |  |  |  |  |  |  |  |  |  |
| 27_2013JUV22 | F | RR | | 115 | 127 | | 223 | | 239 | 98 | | 96 | | 85 | 113 | | 0 | 0 | | 178 | 178 | 187 | | 189 |  | |  |  |  |  |  |  |  |  |  |  |
| 27_2013JUV23 | F | RR | | 117 | 127 | | 0 | | 0 | 98 | | 96 | | 85 | 113 | | 0 | 0 | | 178 | 178 | 187 | | 189 |  | |  |  |  |  |  |  |  |  |  |  |
| 27_2013JUV24 | F | RR | | 117 | 127 | | 223 | | 239 | 98 | | 96 | | 85 | 113 | | 0 | 0 | | 178 | 178 | 187 | | 189 |  | |  |  |  |  |  |  |  |  |  |  |
| 27_2013JUV25 | F | RR | | 117 | 127 | | 223 | | 239 | 98 | | 96 | | 85 | 113 | | 0 | 0 | | 178 | 178 | 187 | | 189 |  | |  |  |  |  |  |  |  |  |  |  |
| 27_2013JUV26 | F | RR | | 115 | 127 | | 0 | | 0 | 98 | | 96 | | 85 | 113 | | 0 | 0 | | 178 | 178 | 187 | | 189 |  | |  |  |  |  |  |  |  |  |  |  |
| 27_2013JUV27 | F | RR | | 115 | 127 | | 0 | | 0 | 98 | | 96 | | 85 | 113 | | 0 | 0 | | 178 | 178 | 187 | | 189 |  | |  |  |  |  |  |  |  |  |  |  |
| 27_2013JUV28 | F | RR | | 115 | 127 | | 0 | | 0 | 98 | | 96 | | 85 | 113 | | 0 | 0 | | 178 | 178 | 187 | | 189 |  | |  |  |  |  |  |  |  |  |  |  |
| 27_2013JUV2 | M | RL | | 117 | 0 | | 223 | | 0 | 96 | | 83 | | 113 | 0 | | 131 | 0 | | 178 | 0 | 197 | | 0 |  | |  |  |  |  |  |  |  |  |  |  |
| 27_2013JUV3 | M | RL | | 117 | 0 | | 223 | | 0 | 96 | | 83 | | 113 | 0 | | 135 | 0 | | 178 | 0 | 197 | | 0 |  | |  |  |  |  |  |  |  |  |  |  |
| 27_2013JUV4 | M | RL | | 117 | 0 | | 223 | | 0 | 96 | | 83 | | 113 | 0 | | 131 | 0 | | 178 | 0 | 197 | | 0 |  | |  |  |  |  |  |  |  |  |  |  |
| 27_2013JUV5 | M | RL | | 117 | 0 | | 223 | | 0 | 96 | | 83 | | 113 | 0 | | 135 | 0 | | 178 | 0 | 197 | | 0 |  | |  |  |  |  |  |  |  |  |  |  |
| 27_2013JUV6 | M | RL | | 117 | 0 | | 223 | | 0 | 96 | | 83 | | 113 | 0 | | 135 | 0 | | 178 | 0 | 197 | | 0 |  | |  |  |  |  |  |  |  |  |  |  |
| 27_2013JUV7 | M | RL | | 115 | 0 | | 223 | | 0 | 96 | | 83 | | 113 | 0 | | 131 | 0 | | 178 | 0 | 187 | | 0 |  | |  |  |  |  |  |  |  |  |  |  |
| 27_2013JUV8 | M | RL | | 115 | 0 | | 223 | | 0 | 96 | | 83 | | 113 | 0 | | 131 | 0 | | 178 | 0 | 187 | | 0 |  | |  |  |  |  |  |  |  |  |  |  |
| 27_2013JUV9 | M | RL | | 115 | 0 | | 223 | | 0 | 96 | | 83 | | 113 | 0 | | 135 | 0 | | 178 | 0 | 197 | | 0 |  | |  |  |  |  |  |  |  |  |  |  |
| 13CZ3WF40M | M | RL | | 127 | 0 | | 239 | | 0 | 98 | | 83 | | 85 | 0 | | 135 | 0 | | 178 | 0 | 189 | | 0 |  | |  |  |  |  |  |  |  |  |  |  |
| 13CZ3WF10F | F | RR | | 106 | 115 | | 173 | | 232 | 98 | | 90 | | 113 | 113 | | 135 | 135 | | 178 | 182 | 197 | | 204 |  | |  |  |  |  |  |  |  |  |  |  |
| 30_2013JUV1 | M | RL | | 115 | 0 | | 173 | | 0 | 90 | | 83 | | 113 | 0 | | 135 | 0 | | 182 | 0 | 197 | | 0 |  | |  |  |  |  |  |  |  |  |  |  |
| 30_2013JUV10 | M | RL | | 115 | 0 | | 232 | | 0 | 90 | | 0 | | 0 | 0 | | 135 | 0 | | 178 | 0 | 204 | | 0 |  | |  |  |  |  |  |  |  |  |  |  |
| 30_2013JUV10B | M | RL | | 106 | 0 | | 0 | | 0 | 90 | | 83 | | 0 | 0 | | 135 | 0 | | 0 | 0 | 204 | | 0 |  | |  |  |  |  |  |  |  |  |  |  |
| 30_2013JUV11 | F | RR | | 115 | 0 | | 0 | | 0 | 0 | | 0 | | 0 | 0 | | 135 | 135 | | 0 | 0 | 0 | | 0 |  | |  |  |  |  |  |  |  |  |  |  |
| 30_2013JUV11B | M | RL | | 115 | 0 | | 232 | | 0 | 98 | | 83 | | 0 | 0 | | 135 | 0 | | 182 | 0 | 204 | | 0 |  | |  |  |  |  |  |  |  |  |  |  |
| 30_2013JUV12B | M | RL | | 106 | 0 | | 173 | | 0 | 98 | | 83 | | 0 | 0 | | 135 | 0 | | 0 | 0 | 197 | | 0 |  | |  |  |  |  |  |  |  |  |  |  |
| 30_2013JUV13 | F | RR | | 115 | 0 | | 0 | | 0 | 98 | | 98 | | 0 | 0 | | 135 | 135 | | 0 | 0 | 189 | | 204 |  | |  |  |  |  |  |  |  |  |  |  |
| 30_2013JUV13B | M | RL | | 115 | 0 | | 0 | | 0 | 90 | | 83 | | 0 | 0 | | 135 | 0 | | 0 | 0 | 204 | | 0 |  | |  |  |  |  |  |  |  |  |  |  |
| 30_2013JUV14 | M | RL | | 115 | 0 | | 232 | | 0 | 98 | | 83 | | 0 | 0 | | 135 | 0 | | 182 | 0 | 197 | | 0 |  | |  |  |  |  |  |  |  |  |  |  |
| 30_2013JUV14B | M | RL | | 115 | 0 | | 173 | | 0 | 90 | | 83 | | 113 | 0 | | 135 | 0 | | 178 | 0 | 197 | | 0 |  | |  |  |  |  |  |  |  |  |  |  |
| 30_2013JUV17 | F | RR | | 106 | 127 | | 0 | | 0 | 98 | | 98 | | 85 | 0 | | 135 | 135 | | 0 | 0 | 189 | | 197 |  | |  |  |  |  |  |  |  |  |  |  |
| 30_2013JUV18 | F | RR | | 106 | 127 | | 0 | | 0 | 98 | | 98 | | 85 | 113 | | 135 | 135 | | 0 | 0 | 189 | | 197 |  | |  |  |  |  |  |  |  |  |  |  |
| 30_2013JUV19 | F | RR | | 115 | 127 | | 0 | | 0 | 98 | | 98 | | 85 | 0 | | 135 | 135 | | 0 | 0 | 189 | | 197 |  | |  |  |  |  |  |  |  |  |  |  |
| 30_2013JUV1B | M | RL | | 106 | 0 | | 232 | | 0 | 90 | | 83 | | 113 | 0 | | 135 | 0 | | 0 | 0 | 204 | | 0 |  | |  |  |  |  |  |  |  |  |  |  |
| 30_2013JUV2 | F | RR | | 106 | 127 | | 173 | | 239 | 98 | | 90 | | 113 | 85 | | 135 | 135 | | 178 | 182 | 189 | | 204 |  | |  |  |  |  |  |  |  |  |  |  |
| 30_2013JUV20 | F | RR | | 115 | 127 | | 0 | | 0 | 98 | | 98 | | 0 | 0 | | 135 | 135 | | 0 | 0 | 189 | | 204 |  | |  |  |  |  |  |  |  |  |  |  |
| 30_2013JUV21 | F | RR | | 106 | 127 | | 0 | | 0 | 98 | | 98 | | 85 | 113 | | 135 | 135 | | 0 | 0 | 189 | | 204 |  | |  |  |  |  |  |  |  |  |  |  |
| 30_2013JUV22 | M | RL | | 115 | 0 | | 232 | | 0 | 90 | | 83 | | 0 | 0 | | 135 | 0 | | 0 | 0 | 197 | | 0 |  | |  |  |  |  |  |  |  |  |  |  |
| 30_2013JUV23 | F | RR | | 106 | 127 | | 0 | | 0 | 98 | | 98 | | 85 | 0 | | 135 | 135 | | 0 | 0 | 189 | | 204 |  | |  |  |  |  |  |  |  |  |  |  |
| 30_2013JUV24 | F | RR | | 115 | 127 | | 0 | | 0 | 90 | | 98 | | 0 | 0 | | 135 | 135 | | 0 | 0 | 189 | | 197 |  | |  |  |  |  |  |  |  |  |  |  |
| 30_2013JUV25 | F | RR | | 106 | 127 | | 0 | | 0 | 98 | | 98 | | 85 | 113 | | 135 | 135 | | 0 | 0 | 189 | | 204 |  | |  |  |  |  |  |  |  |  |  |  |
| 30_2013JUV2B | M | RL | | 115 | 0 | | 232 | | 0 | 98 | | 83 | | 0 | 0 | | 135 | 0 | | 182 | 0 | 204 | | 0 |  | |  |  |  |  |  |  |  |  |  |  |
| 30_2013JUV3 | F | RR | | 106 | 0 | | 173 | | 239 | 98 | | 98 | | 85 | 113 | | 135 | 135 | | 178 | 182 | 189 | | 204 |  | |  |  |  |  |  |  |  |  |  |  |
| 30_2013JUV32 | M | RL | | 106 | 0 | | 232 | | 0 | 98 | | 83 | | 113 | 0 | | 135 | 0 | | 182 | 0 | 204 | | 0 |  | |  |  |  |  |  |  |  |  |  |  |
| 30_2013JUV33 | M | RL | | 115 | 0 | | 0 | | 0 | 98 | | 83 | | 113 | 0 | | 135 | 0 | | 0 | 0 | 0 | | 0 |  | |  |  |  |  |  |  |  |  |  |  |
| 30_2013JUV38 | F | RR | | 115 | 127 | | 232 | | 239 | 98 | | 83 | | 0 | 0 | | 135 | 135 | | 0 | 0 | 189 | | 197 |  | |  |  |  |  |  |  |  |  |  |  |
| 30_2013JUV3B | M | RL | | 106 | 0 | | 173 | | 0 | 98 | | 83 | | 113 | 0 | | 135 | 0 | | 182 | 0 | 204 | | 0 |  | |  |  |  |  |  |  |  |  |  |  |
| 30_2013JUV4 | F | RR | | 106 | 0 | | 173 | | 239 | 98 | | 90 | | 85 | 113 | | 135 | 135 | | 178 | 182 | 189 | | 204 |  | |  |  |  |  |  |  |  |  |  |  |
| 30_2013JUV4B | M | RL | | 115 | 0 | | 0 | | 0 | 90 | | 83 | | 0 | 0 | | 135 | 0 | | 0 | 0 | 197 | | 0 |  | |  |  |  |  |  |  |  |  |  |  |
| 30_2013JUV5 | M | RL | | 106 | 0 | | 173 | | 0 | 90 | | 83 | | 0 | 0 | | 135 | 0 | | 182 | 0 | 204 | | 0 |  | |  |  |  |  |  |  |  |  |  |  |
| 30_2013JUV5B | M | RL | | 106 | 0 | | 232 | | 0 | 90 | | 83 | | 113 | 0 | | 135 | 0 | | 0 | 0 | 197 | | 0 |  | |  |  |  |  |  |  |  |  |  |  |
| 30_2013JUV6 | M | RL | | 106 | 0 | | 232 | | 0 | 90 | | 83 | | 0 | 0 | | 135 | 0 | | 182 | 0 | 197 | | 0 |  | |  |  |  |  |  |  |  |  |  |  |
| 30_2013JUV6B | M | RL | | 106 | 0 | | 173 | | 0 | 98 | | 83 | | 113 | 0 | | 135 | 0 | | 182 | 0 | 204 | | 0 |  | |  |  |  |  |  |  |  |  |  |  |
| 30_2013JUV7 | F | RR | | 106 | 127 | | 232 | | 239 | 98 | | 98 | | 0 | 0 | | 135 | 135 | | 0 | 0 | 189 | | 197 |  | |  |  |  |  |  |  |  |  |  |  |
| 30_2013JUV7B | M | RL | | 115 | 0 | | 173 | | 0 | 90 | | 83 | | 0 | 0 | | 135 | 0 | | 178 | 0 | 197 | | 0 |  | |  |  |  |  |  |  |  |  |  |  |
| 30_2013JUV8 | F | RR | | 115 | 127 | | 173 | | 239 | 98 | | 90 | | 0 | 0 | | 135 | 135 | | 0 | 0 | 189 | | 204 |  | |  |  |  |  |  |  |  |  |  |  |
| 30_2013JUV8B | M | RL | | 115 | 0 | | 232 | | 0 | 98 | | 83 | | 113 | 0 | | 135 | 0 | | 0 | 0 | 197 | | 0 |  | |  |  |  |  |  |  |  |  |  |  |
| 30_2013JUV9B | M | RL | | 115 | 0 | | 232 | | 0 | 98 | | 83 | | 113 | 0 | | 135 | 0 | | 182 | 0 | 204 | | 0 |  | |  |  |  |  |  |  |  |  |  |  |
| 30_2013JUV12 | F | RR | | 115 | 127 | | 0 | | 239 | 98 | | 98 | | 85 | 113 | | 135 | 135 | | 178 | 182 | 189 | | 204 |  | |  |  |  |  |  |  |  |  |  |  |
| 30_2013JUV15 | F | RR | | 115 | 127 | | 0 | | 239 | 98 | | 90 | | 85 | 113 | | 0 | 0 | | 178 | 178 | 189 | | 204 |  | |  |  |  |  |  |  |  |  |  |  |
| 30_2013JUV16 | F | RR | | 106 | 127 | | 232 | | 239 | 98 | | 90 | | 85 | 113 | | 0 | 0 | | 178 | 182 | 189 | | 204 |  | |  |  |  |  |  |  |  |  |  |  |
| 30_2013JUV9 | F | RR | | 106 | 127 | | 173 | | 239 | 98 | | 98 | | 85 | 113 | | 135 | 135 | | 178 | 182 | 189 | | 204 |  | |  |  |  |  |  |  |  |  |  |  |
| 13CZ3WF28M | M | RL | | 127 | 0 | | 173 | | 0 | 103 | | 83 | | 113 | 0 | | 137 | 0 | | 163 | 0 | 192 | | 0 |  | |  |  |  |  |  |  |  |  |  |  |
| 13CZ3WF10F | F | RR | | 106 | 115 | | 173 | | 232 | 98 | | 90 | | 113 | 113 | | 135 | 135 | | 178 | 182 | 197 | | 204 |  | |  |  |  |  |  |  |  |  |  |  |
| 32_2013JUV1B | M | RL | | 106 | 0 | | 173 | | 0 | 90 | | 83 | | 113 | 0 | | 0 | 0 | | 182 | 0 | 204 | | 0 |  | |  |  |  |  |  |  |  |  |  |  |
| 32_2013JUV1 | M | RL | | 115 | 0 | | 173 | | 0 | 98 | | 83 | | 113 | 0 | | 0 | 0 | | 182 | 0 | 204 | | 0 |  | |  |  |  |  |  |  |  |  |  |  |
| 32_2013JUV2 | M | RL | | 115 | 0 | | 173 | | 0 | 90 | | 83 | | 113 | 0 | | 0 | 0 | | 182 | 0 | 204 | | 0 |  | |  |  |  |  |  |  |  |  |  |  |
| 13CZ3WF31M | M | RL | | 117 | 0 | | 173 | | 0 | 98 | | 83 | | 113 | 0 | | 142 | 0 | | 182 | 0 | 189 | | 0 |  | |  |  |  |  |  |  |  |  |  |  |
| 13CZ3WF11F | F | RR | | 115 | 115 | | 173 | | 204 | 0 | | 80 | | 85 | 113 | | 135 | 135 | | 169 | 182 | 192 | | 192 |  | |  |  |  |  |  |  |  |  |  |  |
| 39_2013JUV10 | F | RR | | 117 | 0 | | 173 | | 173 | 98 | | 80 | | 113 | 85 | | 0 | 0 | | 182 | 182 | 189 | | 192 |  | |  |  |  |  |  |  |  |  |  |  |
| 39_2013JUV11 | F | RR | | 117 | 115 | | 173 | | 204 | 0 | | 0 | | 113 | 113 | | 0 | 0 | | 182 | 169 | 189 | | 192 |  | |  |  |  |  |  |  |  |  |  |  |
| 39_2013JUV13 | F | RR | | 117 | 115 | | 173 | | 204 | 98 | | 80 | | 113 | 113 | | 0 | 0 | | 182 | 169 | 189 | | 192 |  | |  |  |  |  |  |  |  |  |  |  |
| 39_2013JUV14 | F | RR | | 117 | 0 | | 173 | | 204 | 98 | | 80 | | 113 | 85 | | 0 | 0 | | 182 | 182 | 189 | | 192 |  | |  |  |  |  |  |  |  |  |  |  |
| 39_2013JUV15 | F | RR | | 117 | 0 | | 173 | | 204 | 98 | | 80 | | 113 | 85 | | 0 | 0 | | 182 | 169 | 189 | | 192 |  | |  |  |  |  |  |  |  |  |  |  |
| 39_2013JUV16 | F | RR | | 117 | 0 | | 173 | | 204 | 98 | | 80 | | 113 | 113 | | 0 | 0 | | 182 | 182 | 189 | | 192 |  | |  |  |  |  |  |  |  |  |  |  |
| 39_2013JUV17 | F | RR | | 117 | 0 | | 0 | | 0 | 98 | | 80 | | 113 | 113 | | 0 | 0 | | 182 | 182 | 189 | | 192 |  | |  |  |  |  |  |  |  |  |  |  |
| 39_2013JUV18 | F | RR | | 117 | 0 | | 0 | | 0 | 98 | | 80 | | 113 | 113 | | 0 | 0 | | 182 | 169 | 189 | | 192 |  | |  |  |  |  |  |  |  |  |  |  |
| 39_2013JUV19 | F | RR | | 117 | 0 | | 0 | | 0 | 98 | | 80 | | 113 | 113 | | 0 | 0 | | 182 | 169 | 189 | | 192 |  | |  |  |  |  |  |  |  |  |  |  |
| 39_2013JUV1B | F | RR | | 117 | 115 | | 173 | | 173 | 98 | | 80 | | 113 | 85 | | 142 | 135 | | 182 | 182 | 189 | | 192 |  | |  |  |  |  |  |  |  |  |  |  |
| 39_2013JUV1 | F | RR | | 117 | 115 | | 173 | | 204 | 98 | | 80 | | 113 | 85 | | 142 | 135 | | 182 | 169 | 189 | | 192 |  | |  |  |  |  |  |  |  |  |  |  |
| 39_2013JUV20 | F | RR | | 117 | 0 | | 173 | | 204 | 98 | | 80 | | 113 | 113 | | 0 | 0 | | 182 | 182 | 189 | | 192 |  | |  |  |  |  |  |  |  |  |  |  |
| 39_2013JUV2B | F | RR | | 117 | 0 | | 173 | | 204 | 98 | | 80 | | 113 | 85 | | 142 | 135 | | 182 | 182 | 189 | | 192 |  | |  |  |  |  |  |  |  |  |  |  |
| 39_2013JUV2 | F | RR | | 117 | 0 | | 173 | | 173 | 98 | | 80 | | 113 | 85 | | 142 | 135 | | 182 | 182 | 189 | | 192 |  | |  |  |  |  |  |  |  |  |  |  |
| 39_2013JUV3B | F | RR | | 117 | 0 | | 173 | | 173 | 98 | | 80 | | 113 | 113 | | 142 | 135 | | 182 | 182 | 189 | | 192 |  | |  |  |  |  |  |  |  |  |  |  |
| 39_2013JUV4B | F | RR | | 117 | 0 | | 173 | | 173 | 98 | | 80 | | 113 | 85 | | 142 | 135 | | 182 | 182 | 189 | | 192 |  | |  |  |  |  |  |  |  |  |  |  |
| 39_2013JUV5B | F | RR | | 117 | 115 | | 173 | | 204 | 98 | | 80 | | 113 | 85 | | 0 | 0 | | 182 | 182 | 189 | | 192 |  | |  |  |  |  |  |  |  |  |  |  |
| 39_2013JUV6B | F | RR | | 117 | 0 | | 173 | | 204 | 98 | | 80 | | 113 | 113 | | 0 | 0 | | 182 | 182 | 189 | | 192 |  | |  |  |  |  |  |  |  |  |  |  |
| 39_2013JUV7B | F | RR | | 117 | 115 | | 173 | | 204 | 98 | | 80 | | 113 | 113 | | 142 | 135 | | 182 | 169 | 189 | | 192 |  | |  |  |  |  |  |  |  |  |  |  |
| 39_2013JUV8B | F | RR | | 117 | 115 | | 173 | | 204 | 98 | | 80 | | 113 | 85 | | 142 | 135 | | 182 | 169 | 189 | | 192 |  | |  |  |  |  |  |  |  |  |  |  |
| 39_2013JUV9B | F | RR | | 117 | 0 | | 173 | | 173 | 98 | | 80 | | 113 | 85 | | 142 | 135 | | 182 | 169 | 189 | | 192 |  | |  |  |  |  |  |  |  |  |  |  |
| 13CZ3WF38M | M | RL | | 106 | 0 | | 223 | | 0 | 90 | | 83 | | 106 | 0 | | 137 | 0 | | 163 | 0 | 197 | | 0 |  | |  |  |  |  |  |  |  |  |  |  |
| 13CZ3WF11F | F | RR | | 115 | 115 | | 173 | | 204 | 0 | | 80 | | 85 | 113 | | 135 | 135 | | 169 | 182 | 192 | | 192 |  | |  |  |  |  |  |  |  |  |  |  |
| 41_2013JUV10 | M | RL | | 0 | 0 | | 173 | | 0 | 80 | | 83 | | 113 | 0 | | 135 | 0 | | 169 | 0 | 192 | | 0 |  | |  |  |  |  |  |  |  |  |  |  |
| 41_2013JUV11 | M | RL | | 0 | 0 | | 173 | | 0 | 80 | | 83 | | 113 | 0 | | 135 | 0 | | 182 | 0 | 192 | | 0 |  | |  |  |  |  |  |  |  |  |  |  |
| 41_2013JUV12 | M | RL | | 115 | 0 | | 204 | | 0 | 80 | | 83 | | 85 | 0 | | 135 | 0 | | 182 | 0 | 192 | | 0 |  | |  |  |  |  |  |  |  |  |  |  |
| 41_2013JUV13 | M | RL | | 0 | 0 | | 204 | | 0 | 80 | | 83 | | 0 | 0 | | 135 | 0 | | 169 | 0 | 192 | | 0 |  | |  |  |  |  |  |  |  |  |  |  |
| 41_2013JUV14 | M | RL | | 115 | 0 | | 173 | | 0 | 80 | | 83 | | 0 | 0 | | 135 | 0 | | 182 | 0 | 192 | | 0 |  | |  |  |  |  |  |  |  |  |  |  |
| 41_2013JUV15 | M | RL | | 0 | 0 | | 204 | | 0 | 80 | | 83 | | 0 | 0 | | 135 | 0 | | 169 | 0 | 192 | | 0 |  | |  |  |  |  |  |  |  |  |  |  |
| 41_2013JUV16 | M | RL | | 0 | 0 | | 173 | | 0 | 0 | | 0 | | 85 | 0 | | 0 | 0 | | 169 | 0 | 192 | | 0 |  | |  |  |  |  |  |  |  |  |  |  |
| 41_2013JUV17 | M | RL | | 0 | 0 | | 173 | | 0 | 0 | | 0 | | 85 | 0 | | 0 | 0 | | 182 | 0 | 192 | | 0 |  | |  |  |  |  |  |  |  |  |  |  |
| 41_2013JUV18 | M | RL | | 0 | 0 | | 204 | | 0 | 0 | | 0 | | 85 | 0 | | 0 | 0 | | 169 | 0 | 192 | | 0 |  | |  |  |  |  |  |  |  |  |  |  |
| 41_2013JUV19 | M | RL | | 0 | 0 | | 0 | | 0 | 0 | | 0 | | 0 | 0 | | 0 | 0 | | 0 | 0 | 0 | | 0 |  | |  |  |  |  |  |  |  |  |  |  |
| 41_2013JUV20 | M | RL | | 115 | 0 | | 204 | | 0 | 0 | | 0 | | 113 | 0 | | 0 | 0 | | 169 | 0 | 192 | | 0 |  | |  |  |  |  |  |  |  |  |  |  |
| 41_2013JUV9 | M | RL | | 115 | 0 | | 204 | | 0 | 80 | | 83 | | 85 | 0 | | 135 | 0 | | 169 | 0 | 192 | | 0 |  | |  |  |  |  |  |  |  |  |  |  |
| 41_2013JUV1 | M | RL | | 115 | 0 | | 204 | | 0 | 80 | | 83 | | 113 | 0 | | 135 | 0 | | 182 | 0 | 192 | | 0 |  | |  |  |  |  |  |  |  |  |  |  |
| 41_2013JUV2 | M | RL | | 0 | 0 | | 173 | | 0 | 80 | | 83 | | 113 | 0 | | 135 | 0 | | 182 | 0 | 192 | | 0 |  | |  |  |  |  |  |  |  |  |  |  |
| 41_2013JUV3 | M | RL | | 115 | 0 | | 173 | | 0 | 80 | | 83 | | 85 | 0 | | 135 | 0 | | 182 | 0 | 192 | | 0 |  | |  |  |  |  |  |  |  |  |  |  |
| 41_2013JUV4 | M | RL | | 115 | 0 | | 204 | | 0 | 80 | | 83 | | 113 | 0 | | 135 | 0 | | 182 | 0 | 192 | | 0 |  | |  |  |  |  |  |  |  |  |  |  |
| 41_2013JUV5 | M | RL | | 115 | 0 | | 204 | | 0 | 80 | | 83 | | 85 | 0 | | 135 | 0 | | 182 | 0 | 192 | | 0 |  | |  |  |  |  |  |  |  |  |  |  |
| 41_2013JUV6 | M | RL | | 0 | 0 | | 173 | | 0 | 80 | | 83 | | 85 | 0 | | 135 | 0 | | 182 | 0 | 192 | | 0 |  | |  |  |  |  |  |  |  |  |  |  |
| 41_2013JUV7 | M | RL | | 115 | 0 | | 173 | | 0 | 80 | | 83 | | 85 | 0 | | 135 | 0 | | 182 | 0 | 192 | | 0 |  | |  |  |  |  |  |  |  |  |  |  |
| 41_2013JUV8 | M | RL | | 0 | 0 | | 204 | | 0 | 80 | | 83 | | 113 | 0 | | 135 | 0 | | 182 | 0 | 192 | | 0 |  | |  |  |  |  |  |  |  |  |  |  |
| 13CZ3WF32M | M | RL | | 119 | 0 | | 0 | | 0 | 98 | | 83 | | 130 | 0 | | 135 | 0 | | 178 | 0 | 189 | | 0 |  | |  |  |  |  |  |  |  |  |  |  |
| 13CZ3WF11F | F | RR | | 115 | 115 | | 173 | | 204 | 0 | | 80 | | 85 | 113 | | 135 | 135 | | 169 | 182 | 192 | | 192 |  | |  |  |  |  |  |  |  |  |  |  |
| 42_2013JUV1 | M | RL | | 115 | 0 | | 173 | | 0 | 80 | | 83 | | 113 | 0 | | 135 | 0 | | 169 | 0 | 192 | | 0 |  | |  |  |  |  |  |  |  |  |  |  |
| 42_2013JUV1B | M | RL | | 115 | 0 | | 173 | | 0 | 80 | | 83 | | 113 | 0 | | 135 | 0 | | 182 | 0 | 192 | | 0 |  | |  |  |  |  |  |  |  |  |  |  |
| 42_2013JUV2 | M | RL | | 115 | 0 | | 204 | | 0 | 80 | | 83 | | 113 | 0 | | 135 | 0 | | 169 | 0 | 192 | | 0 |  | |  |  |  |  |  |  |  |  |  |  |
| 42_2013JUV2B | M | RL | | 115 | 0 | | 173 | | 0 | 80 | | 83 | | 113 | 0 | | 135 | 0 | | 182 | 0 | 192 | | 0 |  | |  |  |  |  |  |  |  |  |  |  |
| 42_2013JUV3 | M | RL | | 115 | 0 | | 204 | | 0 | 80 | | 83 | | 85 | 0 | | 135 | 0 | | 182 | 0 | 192 | | 0 |  | |  |  |  |  |  |  |  |  |  |  |
| 42_2013JUV3B | M | RL | | 115 | 0 | | 173 | | 0 | 80 | | 83 | | 113 | 0 | | 135 | 0 | | 182 | 0 | 192 | | 0 |  | |  |  |  |  |  |  |  |  |  |  |
| 42_2013JUV4 | M | RL | | 115 | 0 | | 173 | | 0 | 80 | | 83 | | 85 | 0 | | 135 | 0 | | 169 | 0 | 192 | | 0 |  | |  |  |  |  |  |  |  |  |  |  |
| 42_2013JUV4B | M | RL | | 115 | 0 | | 173 | | 0 | 80 | | 83 | | 85 | 0 | | 135 | 0 | | 182 | 0 | 192 | | 0 |  | |  |  |  |  |  |  |  |  |  |  |
| 42_2013JUV5 | M | RL | | 0 | 0 | | 204 | | 0 | 80 | | 83 | | 113 | 0 | | 135 | 0 | | 182 | 0 | 192 | | 0 |  | |  |  |  |  |  |  |  |  |  |  |
| 42_2013JUV5B | M | RL | | 115 | 0 | | 173 | | 0 | 80 | | 83 | | 0 | 0 | | 135 | 0 | | 169 | 0 | 0 | | 0 |  | |  |  |  |  |  |  |  |  |  |  |
| 42_2013JUV6 | M | RL | | 115 | 0 | | 204 | | 0 | 80 | | 83 | | 85 | 0 | | 135 | 0 | | 169 | 0 | 192 | | 0 |  | |  |  |  |  |  |  |  |  |  |  |
| 42_2013JUV6B | M | RL | | 115 | 0 | | 173 | | 0 | 80 | | 83 | | 113 | 0 | | 135 | 0 | | 169 | 0 | 192 | | 0 |  | |  |  |  |  |  |  |  |  |  |  |
| 42_2013JUV7B | M | RL | | 115 | 0 | | 173 | | 0 | 80 | | 83 | | 85 | 0 | | 135 | 0 | | 182 | 0 | 192 | | 0 |  | |  |  |  |  |  |  |  |  |  |  |
| 42_2013JUV8B | M | RL | | 115 | 0 | | 173 | | 0 | 80 | | 83 | | 113 | 0 | | 135 | 0 | | 169 | 0 | 192 | | 0 |  | |  |  |  |  |  |  |  |  |  |  |
| 42_2013JUV9B | M | RL | | 115 | 0 | | 204 | | 0 | 80 | | 83 | | 85 | 0 | | 135 | 0 | | 182 | 0 | 192 | | 0 |  | |  |  |  |  |  |  |  |  |  |  |
| 13CZ3WF31M | M | RL | | 117 | 0 | | 173 | | 0 | 98 | | 83 | | 113 | 0 | | 142 | 0 | | 182 | 0 | 189 | | 0 |  | |  |  |  |  |  |  |  |  |  |  |
| 13CZ1WF4F | F | RR | | 133 | 113 | | 173 | | 212 | 98 | | 88 | | 113 | 113 | | 135 | 135 | | 163 | 178 | 192 | | 192 |  | |  |  |  |  |  |  |  |  |  |  |
| 49_2013JUV1 | M | RL | | 113 | 0 | | 173 | | 0 | 98 | | 83 | | 0 | 0 | | 135 | 0 | | 178 | 0 | 192 | | 0 |  | |  |  |  |  |  |  |  |  |  |  |
| 49_2013JUV2 | F | RR | | 117 | 133 | | 173 | | 173 | 98 | | 88 | | 113 | 113 | | 142 | 135 | | 163 | 182 | 189 | | 192 |  | |  |  |  |  |  |  |  |  |  |  |
| 13CZ3WF36M | M | RL | | 117 | 0 | | 236 | | 0 | 90 | | 83 | | 85 | 0 | | 137 | 0 | | 184 | 0 | 208 | | 0 |  | |  |  |  |  |  |  |  |  |  |  |
| 13CZ4WF1F | F | RR | | 115 | 106 | | 204 | | 173 | 103 | | 88 | | 113 | 108 | | 137 | 137 | | 182 | 169 | 208 | | 204 |  | |  |  |  |  |  |  |  |  |  |  |
| 53_2013JUV1 | M | RL | | 115 | 0 | | 173 | | 0 | 88 | | 83 | | 85 | 0 | | 137 | 0 | | 182 | 0 | 208 | | 0 |  | |  |  |  |  |  |  |  |  |  |  |
| 53_2013JUV2 | M | RL | | 106 | 0 | | 204 | | 0 | 103 | | 83 | | 108 | 0 | | 137 | 0 | | 182 | 0 | 208 | | 0 |  | |  |  |  |  |  |  |  |  |  |  |
| 13CZ3WF44M | M | RL | | 108 | 0 | | 173 | | 0 | 103 | | 83 | | 130 | 0 | | 129 | 0 | | 182 | 0 | 192 | | 0 |  | |  |  |  |  |  |  |  |  |  |  |
| 13CZ4WF1F | F | RR | | 115 | 106 | | 204 | | 173 | 103 | | 88 | | 113 | 108 | | 137 | 137 | | 182 | 169 | 208 | | 204 |  | |  |  |  |  |  |  |  |  |  |  |
| 54_2013JUV1 | M | RL | | 106 | 0 | | 204 | | 0 | 88 | | 83 | | 0 | 0 | | 137 | 0 | | 169 | 0 | 208 | | 0 |  | |  |  |  |  |  |  |  |  |  |  |
| 54_2013JUV2 | M | RL | | 106 | 0 | | 204 | | 0 | 103 | | 83 | | 113 | 0 | | 137 | 0 | | 0 | 0 | 208 | | 0 |  | |  |  |  |  |  |  |  |  |  |  |
| 13CZ3WF39M | M | RL | | 110 | 0 | | 173 | | 0 | 90 | | 83 | | 130 | 0 | | 137 | 0 | | 182 | 0 | 187 | | 0 |  | |  |  |  |  |  |  |  |  |  |  |
| 13CZ4WF1F | F | RR | | 115 | 106 | | 204 | | 173 | 103 | | 88 | | 113 | 108 | | 137 | 137 | | 182 | 169 | 208 | | 204 |  | |  |  |  |  |  |  |  |  |  |  |
| 57_2013JUV1 | M | RL | | 106 | 0 | | 204 | | 0 | 103 | | 83 | | 0 | 0 | | 137 | 0 | | 0 | 0 | 204 | | 0 |  | |  |  |  |  |  |  |  |  |  |  |
| 57_2013JUV2 | M | RL | | 106 | 0 | | 173 | | 0 | 103 | | 83 | | 108 | 0 | | 137 | 0 | | 182 | 0 | 208 | | 0 |  | |  |  |  |  |  |  |  |  |  |  |
| 57_2013JUV3 | M | RL | | 106 | 0 | | 204 | | 0 | 88 | | 83 | | 0 | 0 | | 137 | 0 | | 0 | 0 | 0 | | 0 |  | |  |  |  |  |  |  |  |  |  |  |
| 13CZ3WF42M | M | RL | | 113 | 0 | | 204 | | 0 | 96 | | 83 | | 113 | 0 | | 137 | 0 | | 163 | 0 | 189 | | 0 |  | |  |  |  |  |  |  |  |  |  |  |
| 13CZ4WF1F | F | RR | | 115 | 106 | | 204 | | 173 | 103 | | 88 | | 113 | 108 | | 137 | 137 | | 182 | 169 | 208 | | 204 |  | |  |  |  |  |  |  |  |  |  |  |
| 59_2013JUV1 | F | RR | | 106 | 113 | | 204 | | 204 | 103 | | 96 | | 113 | 113 | | 137 | 137 | | 163 | 182 | 189 | | 208 |  | |  |  |  |  |  |  |  |  |  |  |
| 59_2013JUV2 | F | RR | | 113 | 115 | | 204 | | 204 | 0 | | 0 | | 108 | 113 | | 0 | 0 | | 163 | 182 | 189 | | 204 |  | |  |  |  |  |  |  |  |  |  |  |
| 59_2013JUV3 | F | RR | | 113 | 115 | | 204 | | 204 | 0 | | 0 | | 113 | 113 | | 0 | 0 | | 163 | 182 | 189 | | 208 |  | |  |  |  |  |  |  |  |  |  |  |
| 13CZ3WF43M | M | RL | | 115 | 0 | | 173 | | 0 | 90 | | 83 | | 85 | 0 | | 135 | 0 | | 163 | 0 | 204 | | 0 |  | |  |  |  |  |  |  |  |  |  |  |
| 13CZ4WF1F | F | RR | | 115 | 106 | | 204 | | 173 | 103 | | 88 | | 113 | 108 | | 137 | 137 | | 182 | 169 | 208 | | 204 |  | |  |  |  |  |  |  |  |  |  |  |
| 60_2013JUV10 | M | RL | | 115 | 0 | | 173 | | 0 | 88 | | 83 | | 108 | 0 | | 137 | 0 | | 169 | 0 | 204 | | 0 |  | |  |  |  |  |  |  |  |  |  |  |
| 60_2013JUV11 | M | RL | | 106 | 0 | | 173 | | 0 | 103 | | 83 | | 113 | 0 | | 137 | 0 | | 169 | 0 | 204 | | 0 |  | |  |  |  |  |  |  |  |  |  |  |
| 60_2013JUV12 | M | RL | | 106 | 0 | | 204 | | 0 | 88 | | 83 | | 108 | 0 | | 137 | 0 | | 169 | 0 | 204 | | 0 |  | |  |  |  |  |  |  |  |  |  |  |
| 60_2013JUV13 | M | RL | | 106 | 0 | | 204 | | 0 | 0 | | 83 | | 113 | 0 | | 135 | 0 | | 182 | 0 | 208 | | 0 |  | |  |  |  |  |  |  |  |  |  |  |
| 60_2013JUV14 | M | RL | | 106 | 0 | | 204 | | 0 | 88 | | 83 | | 113 | 0 | | 137 | 0 | | 182 | 0 | 208 | | 0 |  | |  |  |  |  |  |  |  |  |  |  |
| 60_2013JUV16 | M | RL | | 106 | 0 | | 173 | | 0 | 103 | | 83 | | 113 | 0 | | 137 | 0 | | 169 | 0 | 208 | | 0 |  | |  |  |  |  |  |  |  |  |  |  |
| 60_2013JUV17 | M | RL | | 106 | 0 | | 204 | | 0 | 88 | | 83 | | 108 | 0 | | 137 | 0 | | 169 | 0 | 204 | | 0 |  | |  |  |  |  |  |  |  |  |  |  |
| 60_2013JUV18 | M | RL | | 106 | 0 | | 204 | | 0 | 103 | | 83 | | 113 | 0 | | 0 | 0 | | 182 | 0 | 208 | | 0 |  | |  |  |  |  |  |  |  |  |  |  |
| 60_2013JUV19 | M | RL | | 115 | 106 | | 173 | | 0 | 88 | | 83 | | 108 | 0 | | 0 | 0 | | 182 | 0 | 204 | | 0 |  | |  |  |  |  |  |  |  |  |  |  |
| 60_2013JUV2 | M | RL | | 115 | 0 | | 173 | | 0 | 103 | | 83 | | 113 | 0 | | 137 | 0 | | 169 | 0 | 204 | | 0 |  | |  |  |  |  |  |  |  |  |  |  |
| 60_2013JUV20 | M | RL | | 106 | 0 | | 204 | | 0 | 103 | | 83 | | 113 | 0 | | 0 | 0 | | 182 | 0 | 208 | | 0 |  | |  |  |  |  |  |  |  |  |  |  |
| 60_2013JUV3 | M | RL | | 115 | 0 | | 173 | | 0 | 103 | | 83 | | 113 | 0 | | 137 | 0 | | 169 | 0 | 204 | | 0 |  | |  |  |  |  |  |  |  |  |  |  |
| 60_2013JUV4 | M | RL | | 115 | 0 | | 173 | | 0 | 88 | | 83 | | 113 | 0 | | 137 | 0 | | 169 | 0 | 204 | | 0 |  | |  |  |  |  |  |  |  |  |  |  |
| 60_2013JUV5 | M | RL | | 106 | 0 | | 173 | | 0 | 88 | | 83 | | 113 | 0 | | 137 | 0 | | 182 | 0 | 208 | | 0 |  | |  |  |  |  |  |  |  |  |  |  |
| 60_2013JUV6 | M | RL | | 106 | 0 | | 204 | | 0 | 103 | | 83 | | 113 | 0 | | 137 | 0 | | 182 | 0 | 208 | | 0 |  | |  |  |  |  |  |  |  |  |  |  |
| 60_2013JUV8 | M | RL | | 106 | 0 | | 173 | | 0 | 88 | | 83 | | 113 | 0 | | 137 | 0 | | 182 | 0 | 208 | | 0 |  | |  |  |  |  |  |  |  |  |  |  |
| 60_2013JUV9 | M | RL | | 115 | 0 | | 204 | | 0 | 103 | | 83 | | 108 | 0 | | 137 | 0 | | 182 | 0 | 208 | | 0 |  | |  |  |  |  |  |  |  |  |  |  |
| 60_2013JUV15 | M | RL | | 106 | 0 | | 173 | | 0 | 103 | | 83 | | 113 | 0 | | 137 | 0 | | 182 | 0 | 208 | | 0 |  | |  |  |  |  |  |  |  |  |  |  |
| 60_2013JUV1 | M | RL | | 106 | 0 | | 173 | | 0 | 103 | | 83 | | 113 | 0 | | 137 | 0 | | 182 | 0 | 204 | | 0 |  | |  |  |  |  |  |  |  |  |  |  |
| 13CZ3WF44M | M | RL | | 108 | 0 | | 173 | | 0 | 103 | | 83 | | 130 | 0 | | 129 | 0 | | 182 | 0 | 192 | | 0 |  | |  |  |  |  |  |  |  |  |  |  |
| 13CZ3WF3F | F | RR | | 106 | 122 | | 173 | | 239 | 98 | | 96 | | 85 | 106 | | 137 | 142 | | 163 | 178 | 189 | | 204 |  | |  |  |  |  |  |  |  |  |  |  |
| 63_2013JUV1 | M | RL | | 122 | 0 | | 239 | | 0 | 96 | | 83 | | 85 | 0 | | 0 | 0 | | 178 | 0 | 189 | | 0 |  | |  |  |  |  |  |  |  |  |  |  |
| 63_2013JUV2 | M | RL | | 122 | 0 | | 239 | | 0 | 96 | | 83 | | 106 | 0 | | 0 | 0 | | 178 | 0 | 189 | | 0 |  | |  |  |  |  |  |  |  |  |  |  |
| 63_2013JUV3 | M | RL | | 122 | 0 | | 239 | | 0 | 96 | | 83 | | 85 | 0 | | 0 | 0 | | 163 | 0 | 204 | | 0 |  | |  |  |  |  |  |  |  |  |  |  |
| 13CZ3WF30M | M | RL | | 137 | 0 | | 204 | | 0 | 77 | | 83 | | 113 | 0 | | 135 | 0 | | 163 | 0 | 192 | | 0 |  | |  |  |  |  |  |  |  |  |  |  |
| 13CZ3WF3F | F | RR | | 106 | 122 | | 173 | | 239 | 98 | | 96 | | 85 | 106 | | 137 | 142 | | 163 | 178 | 189 | | 204 |  | |  |  |  |  |  |  |  |  |  |  |
| 65_2013JUV1 | M | RL | | 106 | 0 | | 239 | | 0 | 96 | | 83 | | 106 | 0 | | 0 | 0 | | 178 | 0 | 189 | | 0 |  | |  |  |  |  |  |  |  |  |  |  |
| 13CZ3WF39M | M | RL | | 110 | 0 | | 173 | | 0 | 90 | | 83 | | 130 | 0 | | 137 | 0 | | 182 | 0 | 187 | | 0 |  | |  |  |  |  |  |  |  |  |  |  |
| 13CZ3WF3F | F | RR | | 106 | 122 | | 173 | | 239 | 98 | | 96 | | 85 | 106 | | 137 | 142 | | 163 | 178 | 189 | | 204 |  | |  |  |  |  |  |  |  |  |  |  |
| 66_2013JUV1 | M | RL | | 122 | 0 | | 239 | | 0 | 96 | | 83 | | 106 | 0 | | 137 | 0 | | 178 | 0 | 189 | | 0 |  | |  |  |  |  |  |  |  |  |  |  |
| 66_2013JUV2 | M | RL | | 122 | 0 | | 173 | | 0 | 98 | | 83 | | 85 | 0 | | 0 | 0 | | 163 | 0 | 189 | | 0 |  | |  |  |  |  |  |  |  |  |  |  |
| 66_2013JUV3 | M | RL | | 106 | 0 | | 173 | | 0 | 98 | | 83 | | 106 | 0 | | 137 | 0 | | 163 | 0 | 204 | | 0 |  | |  |  |  |  |  |  |  |  |  |  |
| 66_2013JUV4 | M | RL | | 106 | 0 | | 173 | | 0 | 96 | | 83 | | 106 | 0 | | 137 | 0 | | 178 | 0 | 189 | | 0 |  | |  |  |  |  |  |  |  |  |  |  |
| 66_2013JUV5 | M | RL | | 122 | 0 | | 239 | | 0 | 98 | | 83 | | 106 | 0 | | 137 | 0 | | 178 | 0 | 189 | | 0 |  | |  |  |  |  |  |  |  |  |  |  |
| 66_2013JUV6 | M | RL | | 106 | 0 | | 239 | | 0 | 0 | | 86 | | 85 | 0 | | 137 | 0 | | 163 | 0 | 204 | | 0 |  | |  |  |  |  |  |  |  |  |  |  |
| 66_2013JUV7 | M | RL | | 122 | 0 | | 239 | | 0 | 96 | | 83 | | 106 | 0 | | 0 | 0 | | 178 | 0 | 189 | | 0 |  | |  |  |  |  |  |  |  |  |  |  |
| 66_2013JUV10 | M | RL | | 106 | 0 | | 239 | | 0 | 98 | | 83 | | 106 | 0 | | 137 | 0 | | 178 | 0 | 189 | | 0 |  | |  |  |  |  |  |  |  |  |  |  |
| 66_2013JUV11 | M | RL | | 122 | 0 | | 239 | | 0 | 96 | | 83 | | 106 | 0 | | 0 | 0 | | 178 | 0 | 189 | | 0 |  | |  |  |  |  |  |  |  |  |  |  |
| 66_2013JUV12 | M | RL | | 122 | 0 | | 173 | | 0 | 98 | | 83 | | 85 | 0 | | 0 | 0 | | 178 | 0 | 189 | | 0 |  | |  |  |  |  |  |  |  |  |  |  |
| 66_2013JUV13 | M | RL | | 122 | 0 | | 173 | | 0 | 96 | | 83 | | 85 | 0 | | 0 | 0 | | 163 | 0 | 204 | | 0 |  | |  |  |  |  |  |  |  |  |  |  |
| 66_2013JUV14 | M | RL | | 122 | 0 | | 173 | | 0 | 98 | | 83 | | 106 | 0 | | 137 | 0 | | 178 | 0 | 189 | | 0 |  | |  |  |  |  |  |  |  |  |  |  |
| 66_2013JUV15 | M | RL | | 122 | 0 | | 173 | | 0 | 98 | | 83 | | 106 | 0 | | 137 | 0 | | 178 | 0 | 189 | | 0 |  | |  |  |  |  |  |  |  |  |  |  |
| 66_2013JUV16 | M | RL | | 122 | 0 | | 173 | | 0 | 98 | | 83 | | 106 | 0 | | 0 | 0 | | 178 | 0 | 189 | | 0 |  | |  |  |  |  |  |  |  |  |  |  |
| 66_2013JUV17 | M | RL | | 122 | 0 | | 173 | | 0 | 98 | | 83 | | 106 | 0 | | 137 | 0 | | 163 | 0 | 189 | | 0 |  | |  |  |  |  |  |  |  |  |  |  |
| 66_2013JUV18 | M | RL | | 122 | 0 | | 239 | | 0 | 98 | | 83 | | 106 | 0 | | 137 | 0 | | 178 | 0 | 204 | | 0 |  | |  |  |  |  |  |  |  |  |  |  |
| 66_2013JUV19 | M | RL | | 106 | 0 | | 239 | | 0 | 98 | | 83 | | 85 | 0 | | 137 | 0 | | 163 | 0 | 204 | | 0 |  | |  |  |  |  |  |  |  |  |  |  |
| 66_2013JUV20 | M | RL | | 106 | 0 | | 239 | | 0 | 96 | | 83 | | 85 | 0 | | 137 | 0 | | 178 | 0 | 189 | | 0 |  | |  |  |  |  |  |  |  |  |  |  |
| 66_2013JUV21 | M | RL | | 122 | 0 | | 239 | | 0 | 98 | | 83 | | 106 | 0 | | 0 | 0 | | 178 | 0 | 189 | | 0 |  | |  |  |  |  |  |  |  |  |  |  |
| 66_2013JUV22 | M | RL | | 122 | 0 | | 173 | | 0 | 98 | | 83 | | 85 | 0 | | 137 | 0 | | 163 | 0 | 204 | | 0 |  | |  |  |  |  |  |  |  |  |  |  |
| 66_2013JUV8 | M | RL | | 122 | 0 | | 239 | | 0 | 96 | | 83 | | 85 | 0 | | 137 | 0 | | 163 | 0 | 189 | | 0 |  | |  |  |  |  |  |  |  |  |  |  |
| 66_2013JUV9 | M | RL | | 122 | 0 | | 173 | | 0 | 0 | | 0 | | 106 | 0 | | 137 | 0 | | 178 | 0 | 189 | | 0 |  | |  |  |  |  |  |  |  |  |  |  |
|  | | |  | | |  | |  | | |  | |  | | |  | | |  | |  | |  | | |  | | |  |  |  |  |  |  |  |  |

Supplementary Table S4. Overwiev on microsatellite loci used in this study. Repeat = minimal and maximal size of the repeat (allele) for each locus. Species-specifity = amplification specifications for RR and LL genomes based on previous studies. RR = *Pelophylax ridibundus*, LL = *P. lessonae*.

|  |  |  | **Repeat** | | **Species-specifity** | |
| --- | --- | --- | --- | --- | --- | --- |
|  | **Locus** | **Label** | **Min.** | **Max.** | **RR** | **LL** |
| **Multiplex 1** | Res20 | red | 106 | 146 | nonamplifying | polymorphic |
|  | RlCA1b5 | yellow | 118 | 138 | polymorphic | polymorphic |
|  | Ga1a19 | blue | 199 | 255 | polymorphic | monomorphic |
|  | RlCA18 | yellow | 169 | 188 | nonamplifying | polymorphic |
|  | RlCA5 | green | 232 | 264 | polymorphic | polymorphic |
|  | RICA2a34 | green | 106 | 160 | polymorphic | polymorphic |
|  | Rrid013A | red | 275 | 299 | polymorphic | polymorphic |
|  | Res14 | blue | 133 | 150 | polymorphic | unknown |
| **Multiplex 2** | Rrid082A | yellow | 161 | 184 | polymorphic | unknown |
|  | Res22 | yellow | 83 | 133 | polymorphic | nonamplifying |
|  | Rrid059A | green | 111 | 139 | polymorphic | monomorphic |
|  | Rrid169A | green | 181 | 214 | polymorphic | nonamplifying |
|  | Re1Caga10 | blue | 97 | 140 | polymorphic | polymorphic |
|  | RlCA1b6 | red | 74 | 108 | polymorphic | polymorphic |
|  | Re2Caga3 | red | 161 | 251 | polymorphic | nonamplifying |

Supplementary Figure S1. Visualization of allele frequencies in R and L genomes of *P. esculentus* sons (RL) and P. ridibundusx daughters (RL) at every family separately. L*essonae* alleles inherited from *P. esculentus* father are in green, *ridibundus* alleles inherited from *P. ridibundus* mother are in red, *ridibundus* alleles inherited from *P. esculentus* father are in blue.


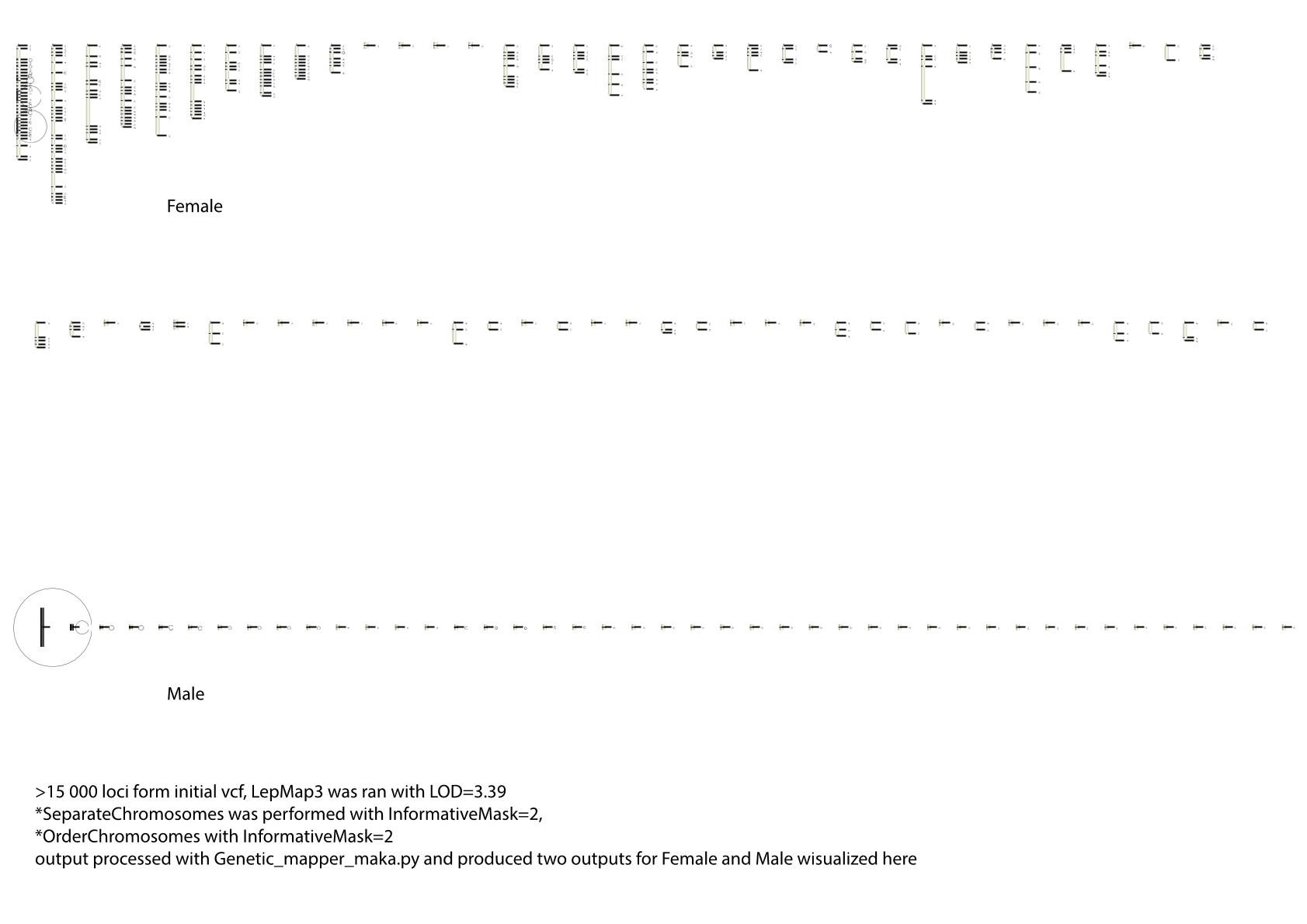


Supplementary Figure S2. Linkage map of 81 individuals as an output processed with Genetic_mapper_maka.py showing two outputs for Female and Male. >15.000 loci form initial vcf, LepMap3 was ran with LOD=3.39, *SeparateChromosomes was performed with InformativeMask=2, *OrderChromosomes with InformativeMask=2.


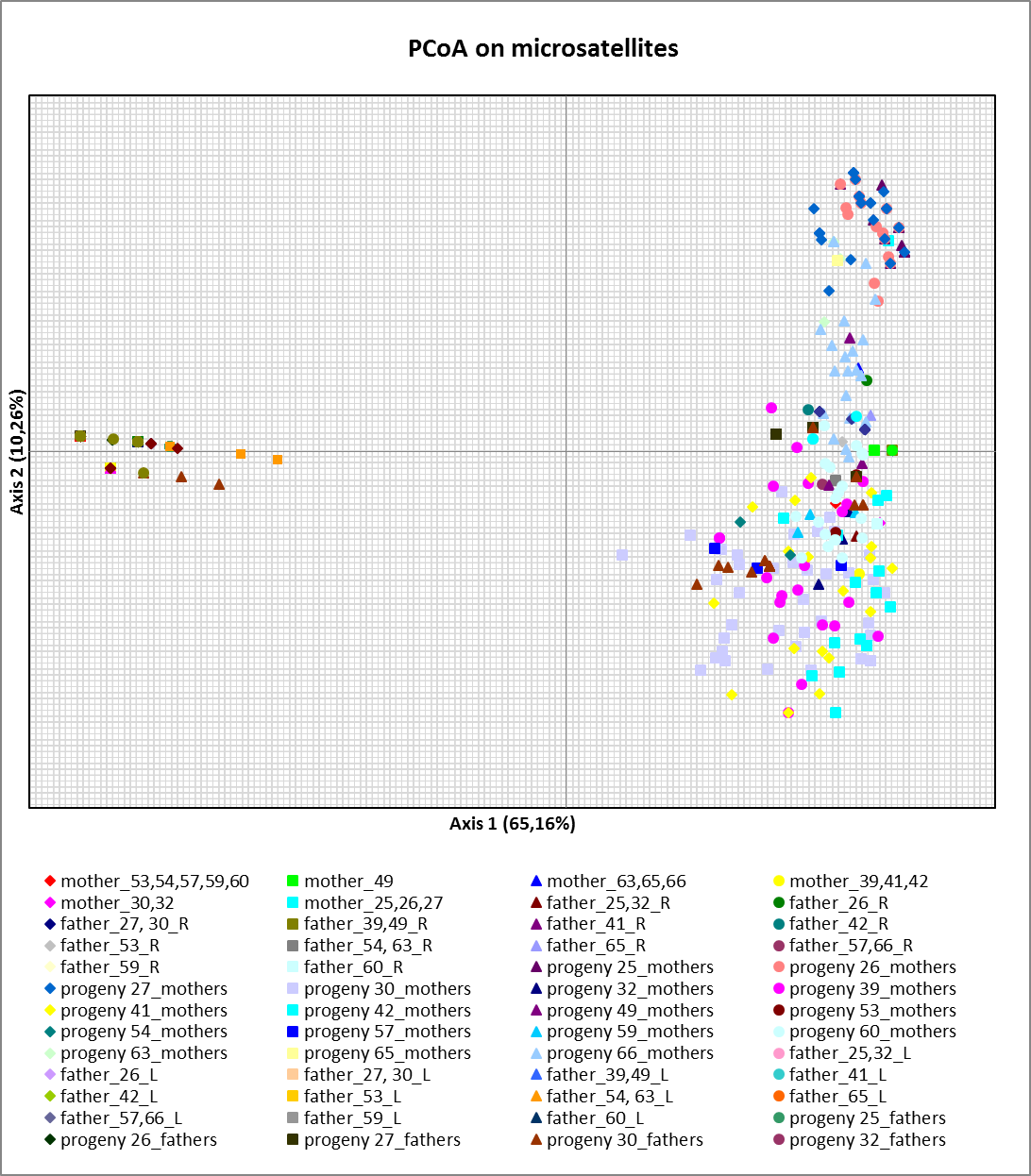

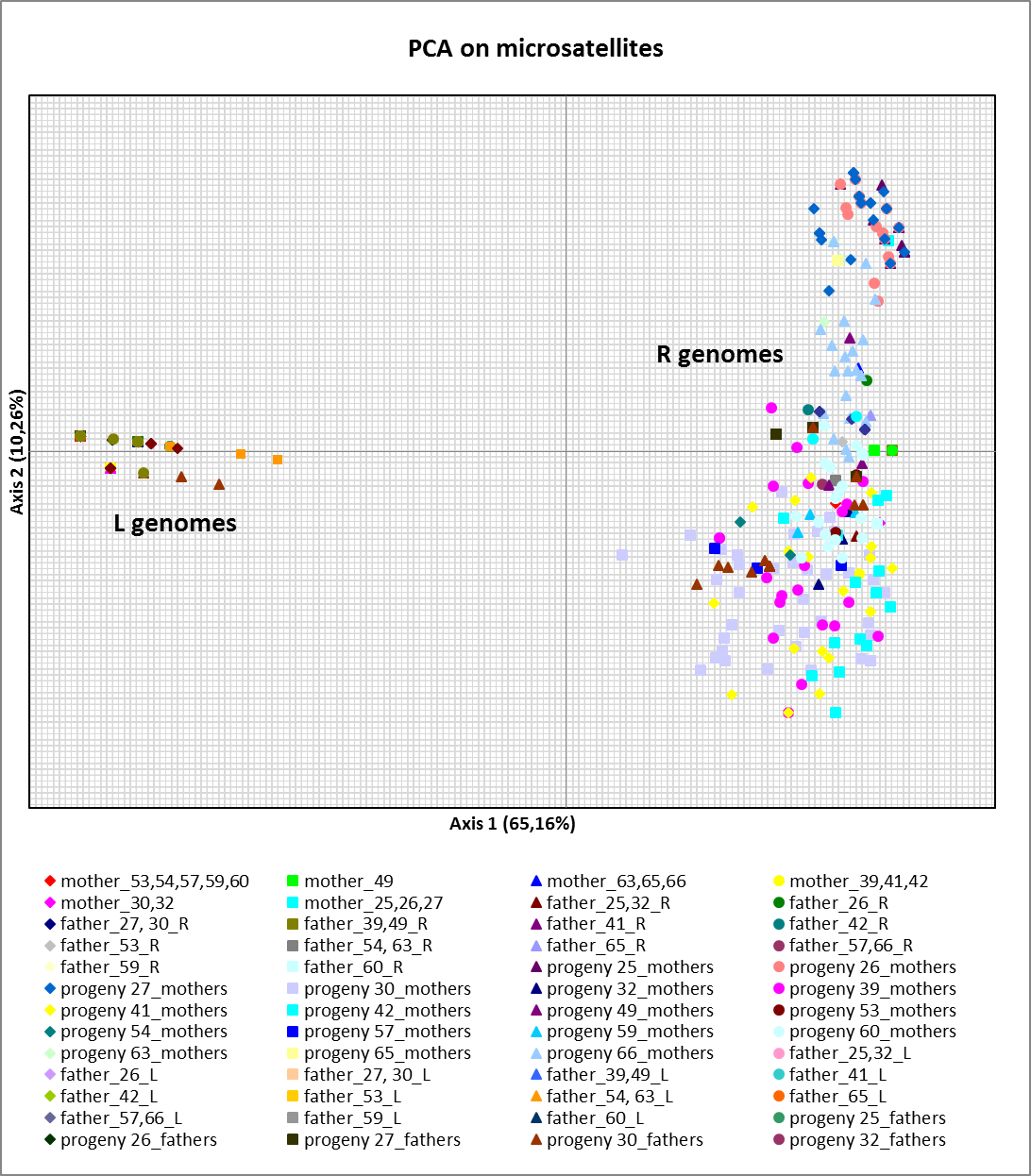


Supplementary Figure S3. Principal Coordinates Analysis (PCoA) of 15 microsatellite loci performed in GenAlEx. Fathers and progeny were phased into haploid MLGs. Each color and symbol individually mark fathers, mothers and respective progeny from a given family. *Lessonae* genomes (L) are on the left, *ridibundus* genomes (R) are on the right . MLGs with more than 65% of missing data were removed from the analysis.


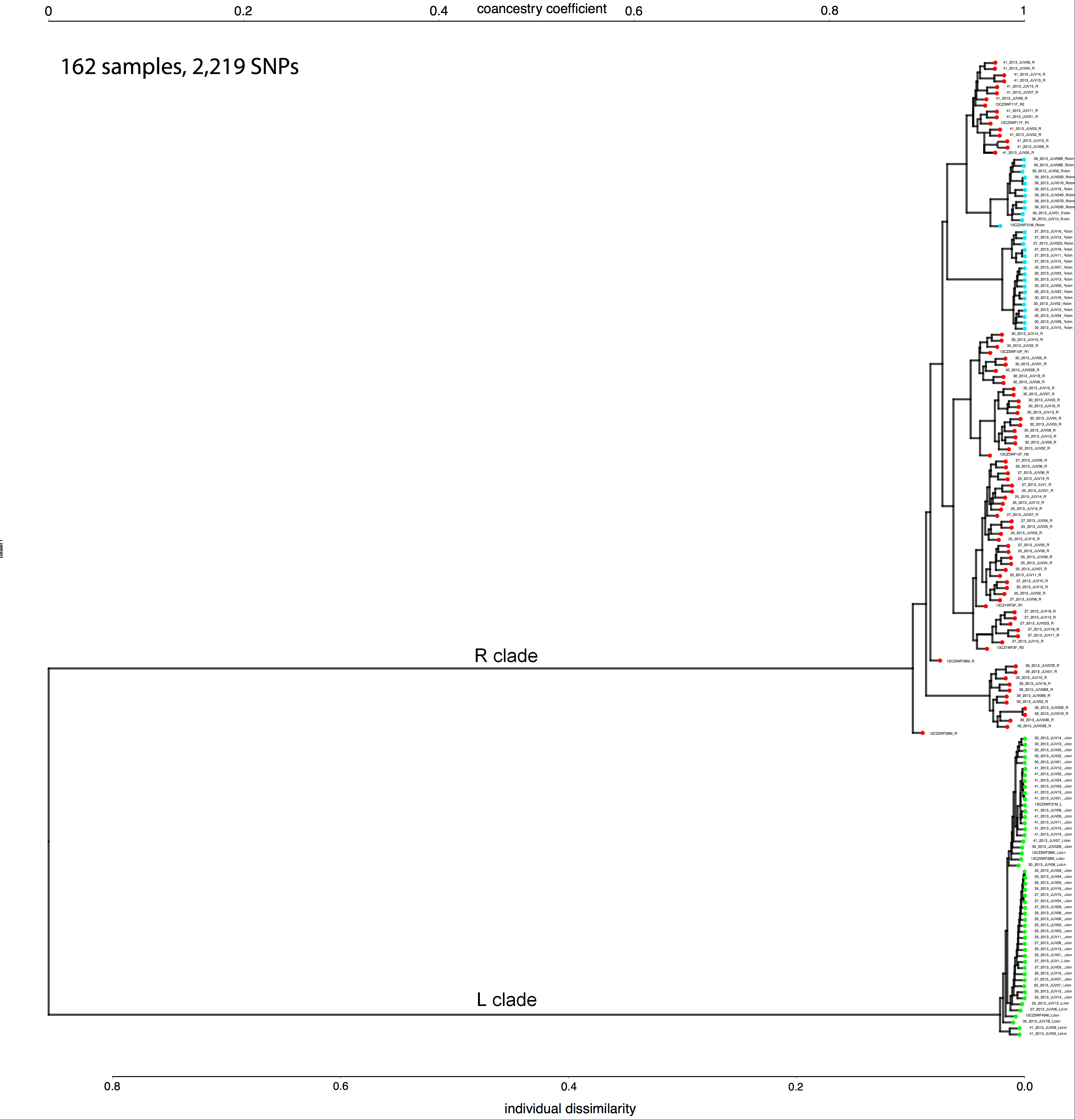


Supplementary Figure S4. The dendrogram of phased haplotypes based on 2’219 SNPs. R clade = *P. ridibundus* like haplotypes, L clade = *P. lessonae* like haplotypes, green color (L clone) = clonal *lessonae* genome inherited from *P. esculentus* fathers, red color (R, R1, R2) = recombined *ridibundus* genome inherited from *P. ridibundus* mothers, blue color = clonal *ridibundus* genome inherited from *P. esculentus* fathers.

Supplementary File S1. Custom script for adapter removal:

cat xxx.fq | paste - - - - | tr '\t' 'z' | sed 's/AGATCz+/z+AGATCz/' | sed 's/AGATCGz+/z+AGATCGz/' | sed 's/AGATCGGz+/z+AGATCGGz/' | sed 's/AGATCGGAz+/z+AGATCGGAz/' | sed 's/AGATCGGAAz+/z+AGATCGGAAz/' | sed 's/AGATCGGAAGz+/z+AGATCGGAAGz/' | sed 's/AGATCGGAAGAz+/z+AGATCGGAAGAz/' | sed 's/AGATCGGAAGAG.*z+z/z+AGATCGGAAGAGz/' | tr 'z' '\t' | awk '{x = length($2); $4 = substr($4,0,x)}1' | tr ' ' '\n' | grep -v '^AGATC.*' | paste - - - - | awk '{x = length($2); $4 = substr($4,0,x)}1' | tr ' ' '\n' > xxx.fq &

#Description: firstly we trnasform each of the 4 lines in the fasq into a single line, with tabs inbetween; start to clip pieces of adaptor that encountered at the end of the sequence and later on the full adapter motif with following sequence, the double occurrences of adaptor-like sequences are handled through grep -v '^AGATC.*' and the last stage is to make equal the length of line 2 (sequence) and 4 (q33 score)

# *Reference genome stitching*

40x coverage, low quality draft genome assembly of *P. lessonae* (C-value based genome size: 6.74 Gb) was produced by Albert Pouska, Jose Grau, and Jörg Plötner (size without N: 4.2 Gb, size including N: 6.3 Gb, N50: 13.6 kb). This assembly is an interleaved fasta file and contains 3’453’250 contigs/scaffolds, while major part of these are very short, 100-150 bp contigs (Figure F1).

Yet, for building the indexes for some mapping software (e.g. bowtie2, STAMPY) or the downstream analyses (e.g. PLINK) this high number of contigs simply cannot be processed, their number can be pushed down by artificial stitching by Ns, selecting longer sequences at the same time. Thus, we chose sequences that were longer 190 bp joining them by 300 Ns.

1) The *Pelophylax lessonae* assembly coming from Jörg Plötner & Co. I made a modification transforming the interleaved fasta into a sequential (one-line) fasta by applying following:

awk '/^>/ {printf("\n%s\n",$0);next; } { printf("%s",$0);}  END {printf("\n");}' < Ples.Jose.fa > Ples.Jose_oneline.fasta &

2) preparation for stitching

#1. We calculate the length for each contig with the python script seq_length.py:

#!/usr/bin/python
from Bio import SeqIO
import sys
cmdargs = str(sys.argv)
for seq_record in SeqIO.parse(str(sys.argv[1]), "fasta"):
 output_line = '%s\t%i' % \
(seq_record.id, len(seq_record))
 print(output_line)

firstly invoking the python script and then running it:

chmod +x seq_length.py
python seq_length.py Ples.Jose_oneline.fasta > Ples.Jose_oneline.length &

Now, we can visualize the distribution of contig length in the genome file with R:

setwd("/Users/gmazepa/UNIX/GENOME_Ples/Ples.Jose_oneline_stats")
ples.full<-read.table(file="length_sorted.txt", sep="\t”)
df<-ples.full$V2
y = count(df)
z<-y[1:100,]
plot(z)


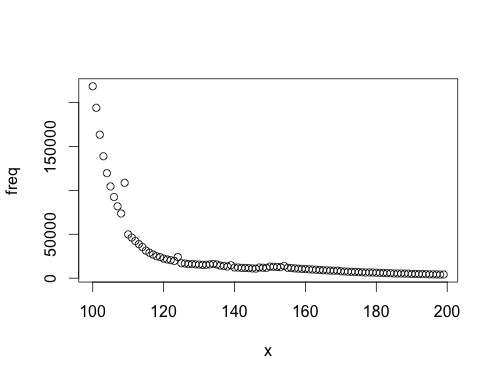


Figure F1. Contig/scaffold size frequencies of the *P. lessonae* draft assembly.

So, after applying the awk script (running in bash mode) for removing short contigs of less than 150 bp:

#!/bin/awk -f
!/^>/ {next}
 {getline s}
length(s) >= i { print $0 "\n" s }
##called by the command:
awk -f removesmalls.sh i=150 input.oneline.fa > output.oneline.longer150.fa

And checking how it did perform (Figure F2):

setwd("/Users/gmazepa/UNIX/GENOME_Ples/Ples.Jose_oneline_stats")
ples.shorter<-read.table(file="Les_Jose.oneline.longer150.length", sep="\t")
df<-ples.shorter$V2
y = count(df)
z<-y[1:100,]
plot(z)


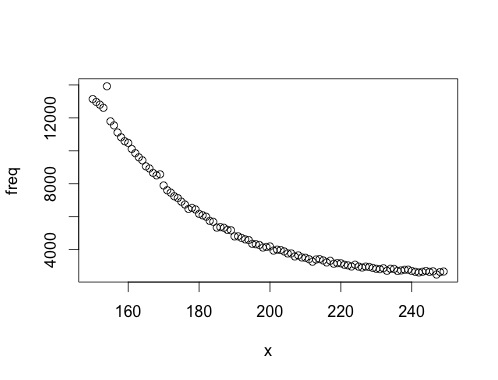


Figure F2. Contig/scaffold size frequencies of the *P. lessonae* draft assembly filtered for sequences >150 bp.

3. Genome stitching.

We can start to stitch the genome using this custom awk script, it will connect each sequential 450 contigs into a supercontig. NB, the first scaffold name won’t be created, the easiest way around this is to firstly create the file with the first line and new line (\n) and only afterwards run the script:

emacs Les_Jose.oneline.longer150.stitched.fa
>scaffold19 15.3\n

Then we are ready to run the stitching script, there are 1’352’557 contigs, so we stitch every 450 to push this number down to ~3000:

# firstly make a first line with the scaffold name in OUTPUT_STITCHED_GENOME.fa, then run
awk '/>/{c++;if(c==450){sub(">","X>");c=0}}1' Les_Jose.oneline.longer150.fa | sed '/^X>/ s/$/X/' | sed -e 's/^>.*/NNNNNNNNNNNNNNNNNNNNNNNNNNNNNNNNNNNNNNNNNNNNNNNNNNNNNNNNNNNNNNNNNNNNNNNNNNNNNNNNNNNNNNNNNNNNNNNNNNNNNNNNNNNNNNNNNNNNNNNNNNNNNNNNNNNNNNNNNNNNNNNNNNNNNNNNNNNNNNNNNNNNNNNNNNNNNNNNNNNNNNNNNNNNNNNNNNNNNNNNNNNNNNNNNNNNNNNNNNNNNNNNNNNNNNNNNNNNNNNNNNNNNNNNNNNNNNNNNNNNNNNNNNNNNNNNNNNNNNNNNNNNNNNNNNNNNNNNNNNN/g' | tr -d '\n' | tr 'X' '\n' >> Les_Jose.oneline.longer150.stitched.fa &

We will end up with the well-stitched genome consisting of quite long super chromosomes:

> max(superchromosome_length)
[1] 14031494
> min(superchromosome_length)
[1] 202200


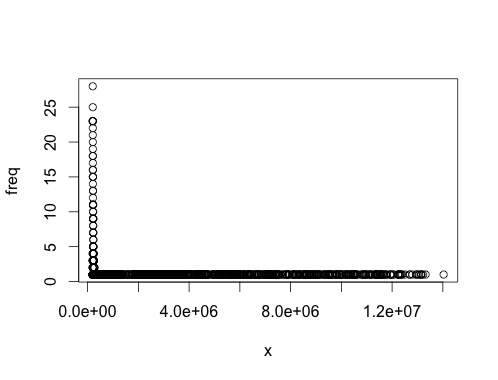


And if we zoom in to the left part of the distribution:


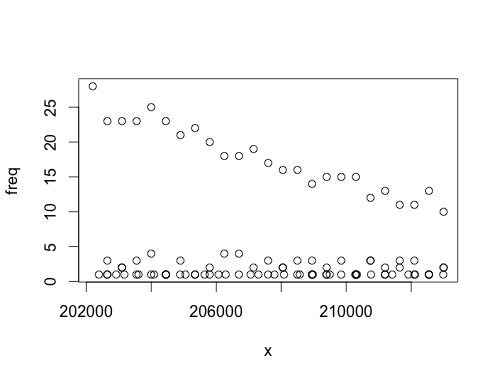


**NB we applied similar procedure to filter out fragments <190 bp and that artificially stitched genome was finally used for mapping of the RADseq data.**

**~~~~~~~~~~~~~~~~~~~~~~~~~~~~~~~~~~~~~~~~~~~~~~~~~~~~~~~~~~~~**

**R code for PCA from the phased VCF**

[**https://github.com/DanJeffries/Hybridogen_paper/tree/master/Data_files**](https://github.com/DanJeffries/Hybridogen_paper/tree/master/Data_files)**/batch_1_PHASED_curated_A_B_C_D_haps.vcf**

library("SNPRelate")

vcf.fn<-"batch_1_PHASED_curated_A_B_C_D_haps.vcf"

snpgdsVCF2GDS(vcf.fn, "ccm.gds", ignore.chr.prefix = "chr")

genofile <- openfn.gds("ccm.gds")

sample.id <- read.gdsn(index.gdsn(genofile, "sample.id"))

# next step - make the list of populations (pops.txt) associated to each indicvid#ual phased haploid genomes (162 in total) according to sample.id order: RR_Mothers, RL_Fathers, Family_25:Father_13CZ3WF28M_R[L]xMother_13CZ1WF3F_RR, Family_27:Father_13CZ3WF40M_[R][L]xMother_13CZ1WF3F_RR, Family_30:Father_13CZ3WF40M_[R][L]xMother_13CZ1WF10F_RR, Family_39:Father_13CZ3WF31M_[R]LxMother_13CZ1WF11F_RR, Family_41:Father_13CZ3WF38M_R[L]xMother_13CZ1WF11F_RR

pop_code <- scan("pops.txt", what=character())

ccm_pca<-snpgdsPCA(genofile, autosome.only=FALSE, num.thread=4)

pc.percent <- ccm_pca$varprop*100

head(round(pc.percent, 2))

tab <- data.frame(sample.id = ccm_pca$sample.id, pop = factor(pop_code)[match(ccm_pca$sample.id, sample.id)], EV1 = ccm_pca$eigenvect[,1], EV2 = ccm_pca$eigenvect[,2],stringsAsFactors = FALSE)

ggplot(data=tab,aes(EV1,EV2, label=tab[,2])) + geom_point(aes(color=as.factor(pop))) + ylab("standardised PC2 (3.10 % explained var.)") + xlab("standardised PC1 (78.6% explained var.)") + scale_color_manual(values=c("darkgoldenrod1" , "chocolate1", "darkorchid1", "burlywood", "red", "chartreuse4", "blue1" )) + theme(aspect.ratio=1)

**Make the SNP-based dendrogram:**

dissMatrix1 = snpgdsIBS(genofile , sample.id=NULL, snp.id=NULL, autosome.only=FALSE, remove.monosnp=TRUE, maf=NaN, missing.rate=0.05, num.thread=2, verbose=TRUE)

snpHCluster = snpgdsHCluster(dissMatrix1, sample.id=NULL, need.mat=TRUE, hang=0.001)

cutTree = snpgdsCutTree(snpHCluster, z.threshold=50, outlier.n=5, n.perm = 5000, samp.group=NULL, col.outlier="red", col.list=NULL, pch.outlier=4, pch.list=NULL,label.H=TRUE, label.Z=TRUE, verbose=TRUE)

snpgdsDrawTree(cutTree, main = "Dataset 2",edgePar=list(col=rgb(0.5,0.5,0.5,1),t.col="black"), y.label.kinship=T,y.label=0.005,leaflab="perpendicular")
